# Evidence for the heterologous benefits of prior BCG vaccination on COVISHIELD™ vaccine-induced immune responses in SARS-CoV-2 seronegative young Indian adults

**DOI:** 10.3389/fimmu.2022.985938

**Published:** 2022-10-04

**Authors:** Srabanti Rakshit, Vasista Adiga, Asma Ahmed, Chaitra Parthiban, Nirutha Chetan Kumar, Pratibha Dwarkanath, Sudarshan Shivalingaiah, Srishti Rao, George D’Souza, Mary Dias, Thomas J. A. Maguire, Katie J. Doores, Martijn Zoodsma, Busranur Geckin, Prokar Dasgupta, Sudhir Babji, Krista E. van Meijgaarden, Simone A. Joosten, Tom H. M. Ottenhoff, Yang Li, Mihai G. Netea, Kenneth D. Stuart, Stephen C. De Rosa, M. Juliana McElrath, Annapurna Vyakarnam

**Affiliations:** ^1^ Centre for Infectious Disease Research, Indian Institute of Science, Bangalore, India; ^2^ Infectious Disease Unit, St. John’s Research Institute, Bangalore, India; ^3^ Department of Biotechnology, PES University, Bangalore, India; ^4^ Division of Nutrition, St. John’s Research Institute, Bangalore, India; ^5^ Department of Pulmonary Medicine, St. John’s Medical College, Bangalore, India; ^6^ Department of Infectious Diseases, School of Immunology and Microbial Sciences, King’s College London, London, United Kingdom; ^7^ Department of Computational Biology for Individualized Infection Medicine, Centre for Individualized Infection Medicine (CiiM), a joint venture between the Helmholtz Centre for Infection Research (HZI) and the Hannover Medical School (MHH), Hannover, Germany; ^8^ TWINCORE, a joint venture between the Helmholtz Centre for Infection Research, (HZI) and the Hannover Medical School (MHH), Hannover, Germany; ^9^ Department of Internal Medicine and Radboud Center for infectious Diseases, Radboud University Medical Center, Nijmegen, Netherlands; ^10^ Peter Gorer Department of Immunobiology, Liver Renal Urology Transplant Gastro/Gastrointestinal Surgery, Inflammation Biology, King’s College London, London, United Kingdom; ^11^ The Wellcome Trust Research Laboratory, Christian Medical College, Vellore, India; ^12^ Department of Infectious Diseases, Leiden University Medical Center, Leiden, Netherlands; ^13^ Centre for Global Infectious Disease Research, Seattle Children’s Research Institute, Seattle, WA, United States; ^14^ Vaccine and Infectious Disease Division, Fred Hutchinson Cancer Research Centre, Seattle, WA, United States; ^15^ Department of Medicine, University of Washington School of Medicine, Seattle, WA, United States; ^16^ Department of Immunobiology, School of Immunology and Microbial Sciences, Faculty of Life Science and Medicine, King’s College London, London, United Kingdom

**Keywords:** BCG, SARS-CoV-2, T cell, antibodies, trained immunity

## Abstract

This proof-of-concept study tested if prior BCG revaccination can qualitatively and quantitively enhance antibody and T-cell responses induced by Oxford/AstraZeneca ChAdOx1nCoV-19 or COVISHIELD™, an efficacious and the most widely distributed vaccine in India. We compared COVISHIELD™ induced longitudinal immune responses in 21 BCG re-vaccinees (BCG-RV) and 13 BCG-non-revaccinees (BCG-NRV), all of whom were BCG vaccinated at birth; latent tuberculosis negative and SARS-CoV-2 seronegative prior to COVISHIELD™ vaccination. Compared to BCG-NRV, BCG-RV displayed significantly higher and persistent spike-specific neutralizing (n) Ab titers and polyfunctional CD4+ and CD8+ T-cells for eight months post COVISHIELD™ booster, including distinct CD4+IFN-γ+ and CD4+IFN-γ- effector memory (EM) subsets co-expressing IL-2, TNF-α and activation induced markers (AIM) CD154/CD137 as well as CD8+IFN-γ+ EM,TEMRA (T cell EM expressing RA) subset combinations co-expressing TNF-α and AIM CD137/CD69. Additionally, elevated nAb and T-cell responses to the Delta mutant in BCG-RV highlighted greater immune response breadth. Mechanistically, these BCG adjuvant effects were associated with elevated markers of trained immunity, including higher IL-1β and TNF-α expression in CD14+HLA-DR+monocytes and changes in chromatin accessibility highlighting BCG-induced epigenetic changes. This study provides first in-depth analysis of both antibody and memory T-cell responses induced by COVISHIELD™ in SARS-CoV-2 seronegative young adults in India with strong evidence of a BCG-induced booster effect and therefore a rational basis to validate BCG, a low-cost and globally available vaccine, as an adjuvant to enhance heterologous adaptive immune responses to current and emerging COVID-19 vaccines.

## Introduction

*Mycobacterium bovis* Bacille Calmette-Guérin (BCG) is administered at birth for tuberculosis (TB) prevention in several TB endemic countries (1). Beyond TB, heterologous non-specific cross-protection benefits of BCG include reducing all-cause mortality and morbidity in infants and children (2, 3) against other pathogens, particularly against viral respiratory infections (4–7). The heterologous benefits of BCG vaccination on adaptive immunity are likely mediated *via* its combined ability to induce Th1/Th17 effectors and humoral responses preceded by an enhanced innate immune response termed ‘trained immunity’ (TI) (8, 9). TI is a biological process by which innate immune responses to pathogen-associated molecular patterns (PAMP) is significantly amplified by prior BCG exposure (10). BCG-induced TI is mediated through epigenetic reprogramming of innate effector genes and cellular metabolism (11, 12), an imprint that can be retained such that subsequent PAMP exposure induces more pronounced innate effector functions in monocytes (13, 14), leading to an innate and adaptive immune response that can be more protective than the one generated in the absence of BCG exposure (10, 15–18).

The COVID-19 outbreak has refocused interest in the cross-protective benefits of BCG in two ways. First, several clinical trials are designed to test if BCG, and indeed more widely available vaccines such as the Influenza, OPV, MMR, Varicella Zoster with reported cross protective benefits, can potentially reduce SARS-CoV-2 infection incidence and disease severity (19–24), with the most compelling protective evidence for BCG emerging from murine live challenge studies (25, 26). Emerging human controlled clinical trial data however, report variable efficacy of BCG vaccination in protecting against SARS-CoV-2 infection (27–30) with rhesus macaques live challenge studies showing no protective effect of BCG (31). Secondly, the known beneficial effects of BCG vaccination on heterologous vaccine responses to Influenza A (HIN1), pneumococcus, tetanus toxoid, measles, mumps, diphtheria, polio (32–36) has prompted exploration of BCG impact on SARS-CoV-2 vaccine-induced immunity. In this context, data from a randomized study in Mexico showed neutralizing antibody (nAb) titers induced by the Pfizer–BioNTech SARS-CoV-2 vaccine to be higher in the group that received BCG first and then the SARS-CoV-2 vaccine relative to the group that received placebo and SARS-CoV-2 vaccine (37). In an animal study, human-ACE2 transgenic mice given BCG coupled with a trimeric-Spike vaccine generated a higher titer of nAb and a greater Th1 response than controls receiving trimeric–Spike vaccine alone and cleared infection with minimal immunopathology following SARS-CoV-2 challenge (25). However, prior exposure to BCG does not guarantee an enhanced adaptive response to all vaccines: Responses to the Vi polysaccharide typhoid fever vaccine (TFV) and the *Haemophilus influenzae* type B (anti-Hib) vaccine was not boosted upon prior BCG exposure (33, 36). Individual vaccines, whether they are live attenuated, whole inactivated or subunit vaccines, can impact the immune system in various ways, and this may or may not necessarily synergize with the non-specific effects of BCG.

These considerations prompted us to explore the impact of prior BCG vaccination on immune responses induced by the Oxford-AstraZeneca ChAdOx1-S, the first SARS-CoV-2 vaccine to be rolled out in India, locally referred to as COVISHIELD™. Our study was exceptionally well positioned to address the above objective: we had initiated a BCG revaccination study of young healthcare workers in October 2019 residing at St. John’s Medical College Hospital, Bangalore with baseline samples (prior to BCG revaccination) and samples at 1 day and 8-10 weeks post BCG revaccination collected before the first COVID-19 outbreak in India. We were therefore in a unique position to track these subjects’ longitudinal responses to the COVISHIELD™ vaccine rolled out in January 2021 relative to their pre-pandemic baseline samples. Our study is the first to provide novel insights into the magnitude and quality of antibody and T cell responses elicited by the COVISHIELD™ vaccine in young Indian adults, who were seronegative against all SARS-CoV-2 proteins screened. This entitles unequivocal analysis of the immune response induced by the COVISHIELD™ priming dose to be placed in context of the COVISHIELD™ booster dose in subjects who did and did not receive prior BCG revaccination.

## Methods

### Ethics statement

This study was performed according to guidelines of the Helsinki Declaration and was approved by the Institutional Ethics Review Committee of St. John Medical College Hospital, Bangalore, IEC Ref no: (IEC/1/896/2018). All study volunteers provided written consent prior to enrolment.

### Study participants

**Figure 1:**
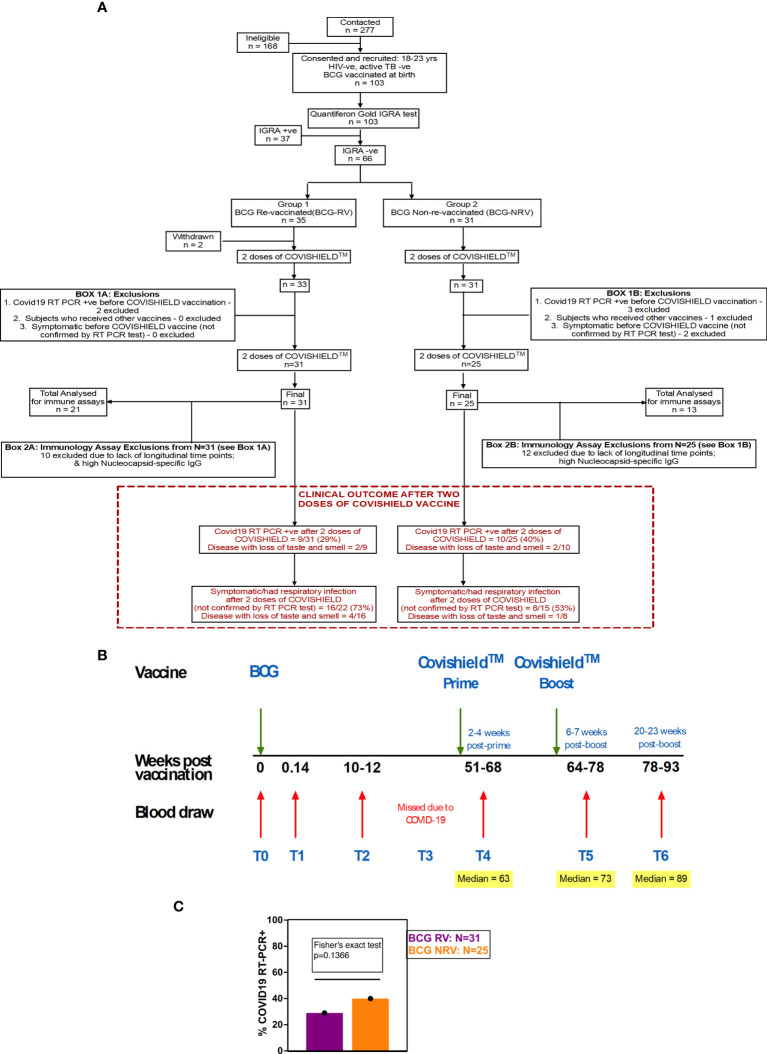
Overall study design. **(A)** CONSORT flow diagram of participant recruitment and enrolment. **(B)** A diagrammatic representation of study design, including schedule of BCG and COVISHIELD™ vaccination and blood draw. Group 1 received BCG at day 0 (T0) and then both groups were vaccinated with 2 doses of COVISHIELD™ vaccine (Prime and Boost). Time points for immunization with BCG and COVISHIELD™ are shown by green arrows, and the 6 blood sampling time points (T0–T6) are indicated by red arrows for all groups. **(C)** Clinical Questionnaire Outcome is shown in red box with bar graph of proportionality data of COVID19 RT-PCR positive BCG RV and BCG NRV subjects.

Healthy healthcare workers aged 18-24 of St. John’s Medical College-Hospital, Bangalore, India were invited to participate in the study from October 2019 to June 2021. All recruited individuals confirmed BCG vaccination at birth. Subjects with chronic illness such as hypertension, diabetes mellitus, heart disease, cancer, kidney/thyroid illness, asthma, epilepsy, jaundice or with a history of clinical tuberculosis disease and on medication were excluded. All participants were assigned a unique serial number. Baseline information included age, gender, medical history, occupation, vaccination status and family history pertaining to tuberculosis. Basic anthropometry measurements of height (cm), weight (kg) was recorded, and body mass index (kg/m^2^) computed. Relevant clinical information of participants is summarized in **Table 1** and detailed follow-up questionnaire is provided in File S1. Blood from participants was screened for Mtb infection by the standard QFT TB Gold In-tube test (Qiagen) performed at Department of Microbiology, SJMCH, India, and were classified as either IGRA+ or IGRA- of which 66 IGRA- subjects were enrolled for the study (**Figure 1A**).

**Table 1:** Clinical table median age for Group 1 and 2 respectively is 20 and 19.

Group	n	Median age (Range)	Male %	Female %	IGRA Level Median (Range)
1: BCG-RV	35	20 (18–22)	54	46	0.06 (0.02-0.57)
2: BCG-NRV	31	19 (18–22)	45	55	0.05 (0.02-0.47)

Male/female ratio in both clinical group is close to 1. There was no significance difference found between male/female IGRA level (QuantiFERON-TB Gold).

### Study design

A prospective observational study was conducted to evaluate the effect of BCG revaccination on subsequent anti-SARS-CoV-2 vaccination. Volunteers were given the choice of either being BCG re-vaccinated (BCG-RV) or not (BCG-NRV) and continue the study protocol of follow-up until 2.6 years. BCG vaccine (TUBERVAC™, Russian BCG strain manufactured at Serum Institute of India, Pune, India), used widely in the Indian national immunization program, was administered intradermally at day 0 at an adult dose of 2 × 10^5^ to 8 × 10^5^ CFU in participants from BCG-RV (n = 35) and BCG-NRV (n = 31) subjects who were not BCG revaccinated served as control. Blood was collected from participants at days 0 (T0), day 1 (T1), 10-12 weeks (T2), 51-68 weeks (T4), 64-77 weeks (T5) and 78-94 weeks (T6) after BCG vaccination (**Figure 1C**). Some BCG vaccinees, reported minor side effects, including itching, rash, or pain at the site of vaccination; mild fever; cough; and headache (**Table S1**). All participants received two doses of COVISHIELD™ vaccine, 7-8 weeks apart, and blood was collected 2-4 weeks post-prime (T4) and either 6-7 weeks (T5) or 20-24 weeks (T6) post-boost (**Figure 1C**). No serious side effects were reported and none of the participants become active TB+ during the entire duration of study. Individuals who turned COVID RT-PCR positive were excluded from downstream assays.

### Peripheral blood mononuclear cell isolation

Blood (16-20 ml) was collected in Na-Heparin tubes (BD, Franklin Lakes NJ, USA) and peripheral blood mononuclear cells (PBMCs) were isolated using 15ml ACCUSPIN (Sigma-Aldrich) tubes by density centrifugation as described previously (38).

### Whole blood ICS assay to track Mtb-specific T cells

Heparinized whole blood was collected from participants and processed within 30-45 min of phlebotomy, as previously described (39). Briefly, 400μl of blood was pipetted into 5ml polypropylene tubes (Sarstedt, Germany) and stimulated with Ag85A peptide pools (1µg/ml per peptide), BCG (0.2 x 10^6^ CFU/ml), or purified recombinant protein ESAT-6/CFP10 (10μg/ml**)** together with anti-CD28/CD49d costimulatory mAbs (0.5µg/ml). Culture medium with anti-CD28/CD49d was used as unstimulated negative control. Blood was incubated at 37˚C for a total of 12hr and Brefeldin A + Monensin (Biolegend) added for the final 10hr of stimulation. After stimulation, blood was processed and archived for future flow cytometry analysis as described previously (38). Please refer **Table 2** for details of antibodies used for staining.

**Table 2:** Antibody panel for innate immunity and to track BCG-specific T cell responses.

Sl. No.	Antibody	Clone	Manufacturer
1	CD14 BV510	M5E2	Biolegend
2	CD16 APC-H7	3G8	BD Pharmingen
3	HLA-DR PE-Cy5	G46-6MQ	BD Pharmingen
4	CD56 BUV737	NCAM16.2	BD Horizon
5	CD3 BV570	UCHT1	Biolegend
6	CD4 BUV393	SK3	BD Horizon
7	CD8 BV711	RPA-T8	BD Horizon
8	IFNg V450	B27	BD
9	IL2 APC	MQ1-17H12	BD Pharmingen
10	TNFα BV605	MAb11	Biolegend
11	IL-1β FITC	AS10	BD
12	IL6 PE-Cy7	MQ2-13A5	Invitrogen

### PBMC ICS assay to track SARS-CoV-2 specific T cell responses

SARS-CoV-2-specific CD4^+^ and CD8^+^ T cell responses were tracked using a validated ICS assay (38). Briefly, cryopreserved PBMC were thawed, and seeded in 96-well round-bottom plates (Costar) at 1 × 10^6^ cells/well in complete RPMI medium {RPMI-1640 (1X) + GlutaMAX™-1 + 25mM HEPES [Invitrogen] supplemented with 10% FCS [Thermo Fisher Scientific], 100 U/mL penicillin and 100μg/mL streptomycin} after 2hr of rest. Cells were stimulated for 20hr at 37°C with Peptivator peptide pools (Miltenyi Biotec) spanning the entire sequence of SARS-CoV-2 structural proteins, i.e., spike (S), nucleocapsid (N) or membrane (M) at a final concentration of 0.06nM in combination with 1 μg/ml aCD28/49d. In selected experiments, cells were also stimulated with the Delta variant (B.1.617.2) and the matched wild-type peptide pool. For negative control, cells were incubated with an equivalent volume of sterile water. CEFT peptide pool (JPT Peptide Technologies) 1μg/ml, and Phytohemagglutinin (PHA, Remel) 4μg/ml, were included as common recall antigen and positive control respectively. Brefeldin A and Monensin (1X, BioLegend) were included in the last 18hr to prevent cytokine release. PBMCs were stained with a panel of antibodies (**Table 3**) by a process described previously (38).

**Table 3:** Antibody panel to track SARS-CoV2 specific T cell responses.

Sl. No.	Antibody	Clone	Manufacturer
1	AviD	–	Molecular Probes
2	CD45RA APC-H7	HI100	BD Pharmingen
3	CCR7 PE-Cy7	G043H7	Biolegend
4	CD56 BUV737	NCAM16.2	BD Horizon
5	CD3 BV570	UCHT1	Biolegend
6	CD4 BUV393	SK3	BD Horizon
7	CD8 BV711	RPA-T8	BD Horizon
8	IFNg V450	B27	BD
9	IL2 Alexa 700	MQ1-17H12	Biolegend
10	TNFα FITC	MAb11	eBioscience
11	IL-17A BV650	N49-653	BD
12	IL-17F BV650	O33-782	BD
13	IL-10 BV786	JES3-9D7	BD
14	CD154 PE-Cy5	24-31	Biolegend
15	CD137 APC	4B4-1	BD
16	CD69 PE	FN50	BD

### Flow cytometry data analysis

Cell fluorescence was acquired on the 5-laser, 18-parameter BD FACSAria™ Fusion flow cytometer (BD Biosciences, San Jose, CA) using BD FACSDiva™ version 8.0.1 software, as previously described (38). Samples were analyzed using FlowJo 10.8.0 (BD Biosciences). Briefly, we have first gated FSC-H vs FSC-A to exclude doublets and then on the singlet gate we have gated out AviD positive cells (FSC-A vs AviD). The gated AviD negative cells have been carried forward for further analysis. All antigen-specific cytokine frequencies are reported after background subtraction of identical gates applied on matched negative controls. Expression of IFN-γ and/or IL-2 was the primary immunogenicity endpoint for CD4+ and CD8+ T cells with an assay cut-off ≥0.02% based on staining of pre-pandemic samples. Background subtractions were performed in Pestle version 1.8. Polyfunctionality of CD4+ cells and CD8+ cells expressing combinations of IFN-γ, IL-2, TNF-α was analyzed with SPICE version 6.1 software (40) as described previously (38). In addition, UMAP, FlowSOM analysis was performed using OMIQ data analysis software (www.omiq.ai). FCS files were uploaded to OMIQ platform along with meta data information. Compensation was performed using single color ultra-comp beads. Cells were sequentially gated into singlets based on FSC-A vs FSC-H > live cells >CD3^+^ and CD3^-^. CD3+ cells are further classified in to CD4+ and CD8+ cells. CD4+ and CD8+ cells from both groups and time points were digitally concatenated and randomly subsampled to 2 million cells where each group and time point has equal numbers of cells. UMAP and FlowSOM was performed on the subsampled CD4+ cells. Edge R was used for statistical tests; 1 and 0.6 million cells were subsampled respectively for the OMIQ analysis.

### PAMP stimulation of PBMC

Cryopreserved PBMC samples were rapidly thawed in a 37°C water bath, transferred to 15ml tubes containing ~ 3ml RPMI+10% FBS and centrifuged at 800g for 5min at RT. A total of 200000 cells resuspended in 200µL culture medium [RPMI-1640 (GIBCO, Invitrogen) supplemented with 10% FBS (GIBCO), 100U/ml penicillin and 100µg/ml streptomycin, (SIGMA)] were seeded per well in 96-well flat-bottom plates (Eppendorf) and stimulated with either 10^6^ cfu/ml of heat-killed *Candida albicans* Strain SC5314 (kind gift from David Moyes; King’s College London), 0.2 x 10^6^ cfu/ml of BCG (TUBERVAC™, Serum Institute of India), 50μg/ml Pam_3_CSK_4_ (Sigma) or 1ng/ml LPS (Sigma). Cells cultured with medium alone were used as negative control. 24hr later plates were centrifuged at 800g for 3min and culture supernatants collected and frozen at -20°C till ELISA was performed.

### ELISA measurements for TNF-α, IL-1β and IL-6

Supernatants from PAMP stimulation cultures were used for measuring levels of TNF-α, IL-1β and IL-6 exactly as per manufacturer’s instructions (TNF-α-BD; IL-1β-Biolegend and IL-6-Biolegend). For each ELISA, assay background was subtracted from absorbance values. Also, the spontaneous cytokine release in cells cultured with medium alone was subtracted from all PAMP stimulation conditions.

### ELISA for anti-Spike, RBD or nucleocapsid IgG

Plasma isolated from heparinized whole blood was used to measure SARS-CoV-2 Spike, RBD or Nucleocapsid specific IgG using an in-house ELISA as previously described (41). Recombinant full-length Spike and RBD for ELISA were expressed and purified as previously described (42). The plasmids for S and RBD were obtained from Philip Brouwer, Marit van Gils and Rogier Sanders at The University of Amsterdam and Florian Krammer at Mount Sinai University (43) respectively. N protein was obtained from Leo James and Jakub Luptak at LMB, Cambridge. It is a truncated construct of the SARS-CoV-2 N protein comprising residues 48-365 (both ordered domains with the native linker) with an N terminal uncleavable hexa-histidine tag.

### Anti-spike IgG measured using the LIAISON^®^ SARS-CoV2 TrimericS IgG assay

The LIAISON^®^ SARS-CoV-2 TrimericS IgG assay (44) (Diasorin.com, accessed on 31st Jan22) was used to measure the anti-spike IgG levels in plasma. This commercial platform is a new generation chemiluminescence immunoassay, using a recombinant Trimeric Spike protein as the capture antigen. The assay has a range of 4.81 to 2080 BAU/ml (Binding Antibody Units/ml). Samples with high titers (>2080 BAU/ml) were diluted further as per the kit manufacturer’s guidelines. The binding antibody units measured in this assay are mapped to the 1st WHO international standard for anti-SARS-CoV-2 immunoglobulin (NIBSC Code-20/136). Any sample below 33.8 BAU/ml is reported as negative for Anti-Spike IgG antibody.

### Viral entry inhibition assay with SARS-CoV2 pseudotyped virus

Pseudotyped HIV virus incorporating the SARS-CoV-2 spike protein [Wuhan-1 and delta (B.1.617.2)] were produced as previously described (45, 46). Serial dilutions of heat-inactivated plasma were prepared with DMEM (10%FBS and 1%Pen/Strep) and incubated with pseudotype virus for 1hr at 37°C in 96-well plates. Next, HeLa cells stably expressing the ACE2 receptor (provided by Dr James Voss, The Scripps Research Institute) were added (10,000 cells/25µL per well) and the plates were left for 72hr. Infection level was assessed in lysed cells with the Bright-Glo luciferase kit (Promega), using a Victor™ X3 multilabel reader (Perkin Elmer). Measurements were performed in duplicates used to calculate the ID50.

### ATAC-seq (assay for transposase-accessible chromatin using sequencing)

Untreated PBMCs were subjected to fragmentation to prepare for ATACseq. The cells were lysed with TDE1 (Tagment DNA enzyme, Illumina). After lysis, DNA fragments were eluted following Qiagen Min Elute kit protocol. DNA was subsequently PCR amplified by using KAPA HiFi Hotstart Ready Mix (KAPA Biosystems) and Nextera Index Kit (Illumina) primers followed by reverse phase 0.65 x SPRI beads purification and a QIAquick Spin Column (QIAGEN) purification. Amplified DNA libraries were sequenced with an Illumina NextSeq 500 at a read length of 38 bp. Bulk ATACseq reads were pre-processed using the publicly available nfcore/atacseq pipeline [(47), v1.2.1] implemented in Nextflow [(48), v21.04.3] using default settings and the human GRCh38 genome. We considered the consensus peak set called by MACS2 in broad mode. Peaks located on the X and Y chromosomes were removed from further consideration, and peaks were annotated to nearby genes using the HOMER toolset (49). Based on PCA-based dimensionality reduction, one sample post BCG vaccination was removed. Differential peak analysis was performed using DESeq2 [(50), v1.30.1], incorporating individual’s donor identifier in the linear model with paired data. Across the peaks, FDR<0.05 was used as a significance threshold. Enrichment of pathways among the open peaks was calculated by considering the genes linked to each peak.

### Statistical analysis

Data were analyzed using FlowJo 10.8.0. Statistical analyzes were performed in GraphPad Prism 9.2, unless otherwise stated. The statistical details of the experiments are provided in the respective figure legends. Data plotted in logarithmic scales were expressed as geometric means ± standard deviations (SD). Mann-Whitney U or Wilcoxon signed-rank t tests were applied for unpaired or paired comparisons, respectively. Differences among longitudinal timepoints were evaluated using Kruskal-Wallis and Dunn’s post-test for multiple comparisons.

## Results

### Study overview

Funded in 2019, this study, was initially designed to probe the efficacy of BCG revaccination on enhancing *Mycobacterium tuberculosis* (Mtb)-specific immune responses, following our previous work (38). Due to the COVID-19 pandemic, we pivoted to probe the impact of BCG revaccination on COVISHIELD™ vaccine-induced immune responses. We recruited 103 young health care workers at St. John’s Medical College Hospital who had all received BCG vaccine at birth, of whom 66 were confirmed Interferon Gamma Release Assay (IGRA) negative (**Table 1**). Of these, 35 received BCG revaccination and 31 did not (see Methods). Samples were collected at baseline (T0), 1 day (T1) and 8-10 weeks-post BCG revaccination (T2); these time points were between September 2019 and January 2020 before COVID-19 pandemic spread to India. Subsequent sample collection (T3) between March 2020 and August 2020 was abandoned. Following roll out of the COVISHIELD™ vaccine in January 2021, we collected samples post COVISHIELD™ vaccination from both BCG re-vaccinees (BCG-RV) and BCG non-vaccinees (BCG-NRV). The median interval between BCG revaccination and the first dose of COVISHIELD™ vaccine was 63 weeks. We collected samples at 2-, 3- or 4-weeks after COVISHIELD™ prime (collectively referred to as T4 prime time point), followed by 5-6 weeks after the booster vaccine (T5 time point), or at 20-23 weeks post-booster (T6 time point). Due to COVID-19 restrictions, subjects were sampled only at one time point post prime and one time point post boost. For COVISHIELD™ vaccine-induced immune data analysis, the following samples were excluded: (i) samples not matched to the above time points, (ii) all subjects who tested SARS-CoV-2 RT-PCR positive, (iii) subjects identified to be potentially exposed to SARS-CoV-2 based on a positive in-house IgG antibody binding assay to the SARS-CoV-2 nucleocapsid (N) protein (see **Figure S1**), and (iv) all dropout subjects where we were unable to collect samples at one of the prime and one of the boost time points. In total, we successfully collected time matched samples from 34 subjects, of which 21 were BCG Re-vaccinees (BCG-RV) and 13 were not re-vaccinated with BCG (BCG-NRV) (**Figure 1**).

A summary of a clinical questionnaire for COVID-19 infection rates in all 31 BCG-RV and 25 BCG-NRV recruited who had all received two doses of COVISHIELD™ vaccine in 2021 is provided. Nine of 31 BCG-RV (29%) and 10 out of 25 BCG-NRV (40%) self-reported to be COVID-19 RT-PCR+ between April 2021 and April 2022 after their second dose of COVISHIELD™ in April 2021 (not statistically significant in a proportionality test) (**Figure 1C**) highlighting that BCG revaccination did not significantly impact COVID-19 infection rates in this study, though a trend for higher infection was noted in BCG-NRV compared to BCG-RV.

### BCG revaccination enhances the magnitude of COVISHIELD™ induced spike-specific immune responses

**Figure 2:**
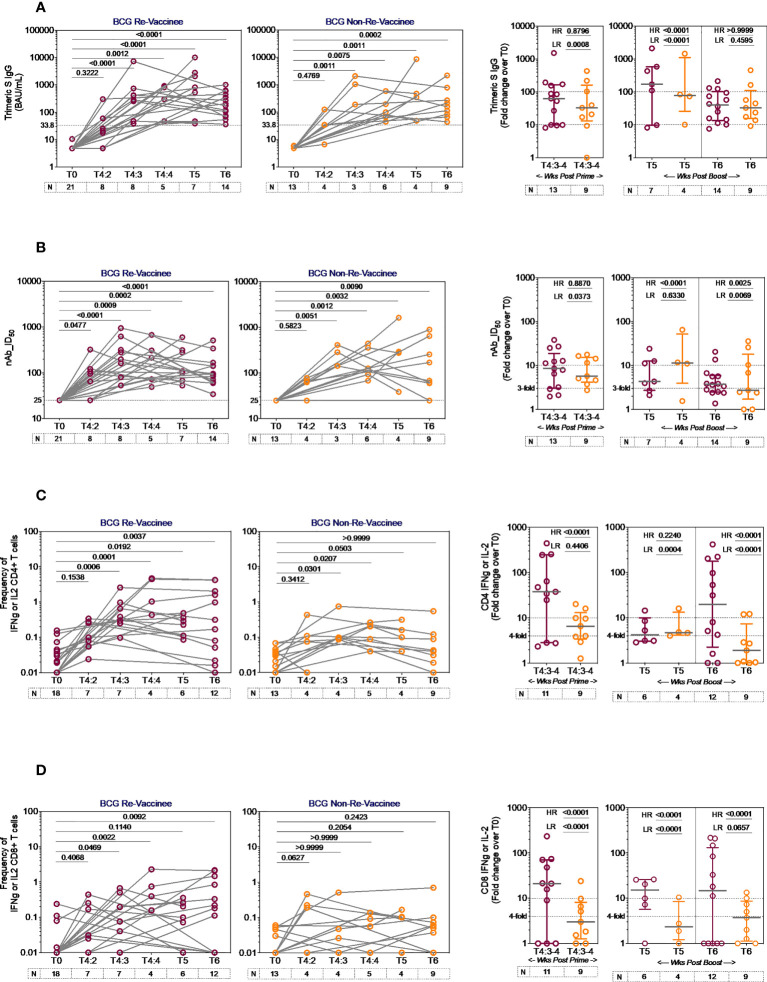
Kinetics of spike-specific vaccine induced responses in BCG-RV and BCG-NRV. Longitudinal analysis of antibody and T cell responses in COVISHIELD™ vaccinated BCG-RV (purple circles) and BCG-NRV (orange circles) subjects at baseline (T0), 2-, 3- and 4-weeks post-prime (T4:2, T4:3 and T4:4), 6-7 weeks post boost (T5) and 20-23 weeks post-boost (T6). **(A)** SARS-CoV-2 anti Spike protein IgG titres measured by LIAISON^®^ SARS-CoV-2 TrimericS IgG assay and **(B)** neutralizing antibody titres (nAb ID_50_) in paired plasma samples. **(C, D)** PBMCs were stimulated with Spike peptide pool (0.06 nM) for 20 hr. CD4+ and CD8+ T cells were analyzed for intracellular expression of IFN-γ or IL-2. Grouped scatter plot with median (horizontal grey line) and interquartile range comparing fold change in plasma antibody titres and frequencies of IFN-γ or IL-2 CD4+ and CD8+ T cells over baseline or at 3-4 weeks post-prime (T4:3-4), 6-7 weeks post boost (T5) and 20-23 weeks post boost (T6). Statistical significance was determined by Kruskal-Wallis test with Dunn’s correction for line graphs and Wilcoxon matched paired t-test for scatter plots. The proportion of each group that showed a positive serologic response to Spike, neutralizing antibody titres or a positive IFN-γ or IL-2 CD4+ and CD8+ T cell response to Spike were compared between COVISHIELD™ vaccinated BCG-RV (purple circles) and BCG-NRV (orange circles) by using Fisher’s exact test. HR indicates the p-value for high-responders in each group (subjects with >100-fold change over baseline for TrimericS IgG, >10-fold change for nAb, CD4+ or CD8+ T-cell responses). LR indicates the p-value for low-responders (>10-fold change for TrimericS IgG, >3-fold change for nAb and >4-fold change for CD4 or CD8 T-cell responses).

The line graphs in **Figure 2** demonstrate the time course of COVISHIELD™ induced Spike specific Ab binding and nAb (**Figures 2A, B** respectively) and CD4+ and CD8+ T-cell responses (**Figures 2C, D** respectively, **Figure S2**) in BCG-RV and BCG-NRV groups. The data highlight three points: (i) Ab kinetics were similar in BCG-RV and BCG-NRV: peak at 3 weeks, dip at 4 weeks post prime and increase at 5-6 weeks post booster with a decline after 20-23 weeks post the booster (**Figures 2A, B**). (ii) CD4+ T-cell responses peaked at 4 weeks post prime with subsequent decline. CD8+ T-cell responses also peaked at 4 weeks post prime noticeable in BCG-RV, with no further increase by the booster vaccine. Instead, a contraction of spike-specific CD4+ and CD8+ T-cell frequencies was noted at both the T5 and T6 time points relative to peak values, with some noticeable differences: median CD4+ and CD8+ T-cell frequencies were higher in BCG-RV versus BCG-NRV, with a trend to remain higher in BCG-RV at 20-23 weeks post boost (**Figures 2C, D**, **Figure S2B**). Furthermore, BCG-NRV CD4+ and CD8+ T-cell responses contracted to near baseline levels at T6, whereas in BCG-RV, the median remained 10-fold higher than the baseline. (iii) The proportion of COVISHIELD™ non-responders (subjects who did not respond to either prime and boost) did not differ between BCG-RV and BCG-NRV. Frequencies of TNF-α+ CD4+ and CD8+ T cells (**Figure S3**) clearly shows that TNF-α is increased for both BCG-RV and BCG-NRV groups at 4 weeks post-prime which is not further enhanced post-boost, however the magnitude of the response is higher in BCG-RV compared to BCG-NRV group.

Comparative analysis of Spike-specific responses in BCG-RV and BCG-NRV was conducted by stratifying responders in time matched samples (**Figure 2** scatter graphs) with a contingency test comparing fold induction, relative to T0 and stratified based on median fold change and spread recorded in each assay. For Ab binding (**Figure 2A**) high and low responses (HR, LR) were set at minimum 100-fold increase over baseline and minimum 10-fold increase over baseline, respectively. For nAb (**Figure 2B**), HR was 10-fold and LR 3-fold. For CD4+ (**Figure 2C**) and CD8+ (**Figure 2D**) T-cell responses, HR was 10-fold and LR 4-fold. In each assay, BCG-RV had a significantly higher proportion of HR and/or LR responders than BCG-NRV (between group comparison minimum p-value=0.0373; maximum p<0.0001). For nAb (**Figure 2B**, scatter graph), the biggest difference was noted 20-23 weeks post booster. For both CD4+ (**Figure 2C**) and CD8+ T cell responses (**Figure 2D**), BCG-RV had a greater proportion of high responders after prime (T4). Taken together, the data highlights BCG-RV subjects to be better and more high responders to COVISHIELD™ than the BCG-NRV subjects. COVISHIELD™ induced T-cell responses were confirmed to be Spike specific with parallel cultures showing absence of T-cell responses to peptide pools covering SARS-CoV-2 M or N and no significant changes to recall antigen CEFT peptides over time (**Figure S4**). Further, COVISHIELD™ induced nAb and T-cell responses (**Figure S5**) showed a significant correlation (*p* range: <0.0069 to <0.0001) that was not strictly linear (r range: 0.4609 to 0.6848) confirming that in both BCG-RV and BCG-NRV subjects, COVISHIELD™ vaccination concurrently induced spike-specific Ab and T-cell responses.

### BCG revaccination significantly enhances the quality of spike-specific T-cell responses

**Figure 3:**
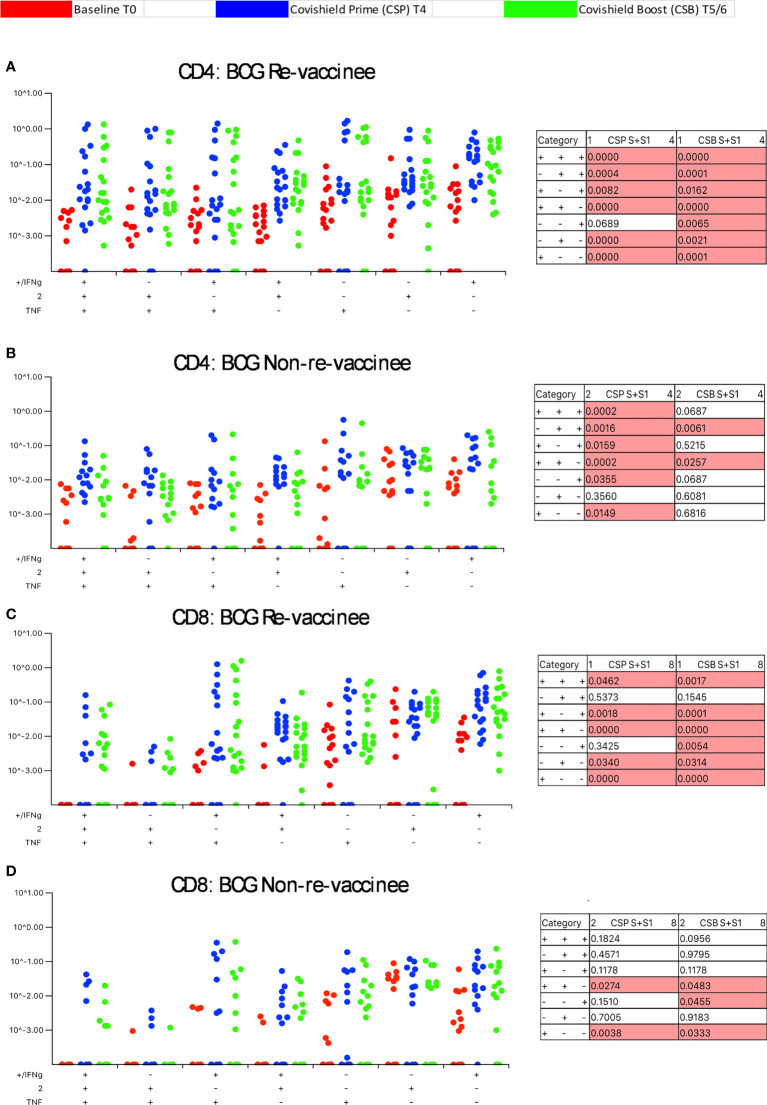
BCG revaccination significantly impacts the quality of the spike-specific CD4+ and CD8+ T cell response in COVISHIELD™-vaccinated subjects. Longitudinal multifunctional spike- specific CD4+ T cells **(A, B)** or CD8+ T cells **(C, D)** in COVISHIELD™ vaccinees. PBMCs from individuals collected at baseline (BL, red dots), 2-4 weeks post-prime (CSP, blue dots) and 6-7- or 20-23-weeks post-boost (CSB, green dots) were stimulated with spike for 20hr and CD4+ or CD8+ T cells were analyzed for intracellular expression of IFN-γ, IL-2 and TNF-α in a standard ICS assay. Boolean gates were created from the individual cytokines (listed above) in FlowJo to divide responding cells into 7 distinct subsets corresponding to all possible combinations of these functions, and the data were analyzed using SPICE software. Data were analyzed for statistical significance using Wilcoxon signed-rank test. Background subtracted and log data analyzed in all cases. P < 0.05 was considered statistically significant.

Our immune-staining panel included 5 effector cytokines (IFN-γ, IL-2, TNF-α, IL-17 and IL-10). Initial analysis demonstrated sporadic expression of IL-17 and IL-10 (data not shown); therefore, downstream analysis focused on IFN-γ, IL-2 and TNF-α effectors. **Figure 3** shows the pattern of seven CD4+ T-cell subsets expressing IFN-γ, IL-2 and TNF-α induced post-prime (blue dots) and post-booster (green dots), relative to matched baseline value (red dots) in BCG-RV (**Figure 3A**) and BCG-NRV (**Figure 3B**) with each dot representing a donor. The matched table lists *p* values calculated by SPICE of the induced response over baseline at prime (CSP) and after boost (CSB). In BCG-RV CD4+ T cell subsets expressing 3, 2 and 1 effectors were significantly induced post-prime, and sustained until 20-23 weeks after the booster (**Figure 3A**). In BCG-NRV, TNF-α+ and IL-2+ single positive (SP) expressing CD4+ T-cells were not induced significantly post-prime (**Figure 3B**), whilst cellular subsets expressing 3, some 2 and some 1 combinations of effectors were. Additionally, in BCG-NRV, only two of the seven subsets analyzed (IL-2/TNF-α double positive {DP} and IFN-γ/IL-2 DP) were sustained post-booster; in particular, cells expressing all 3 cytokines and single effectors were not sustained (**Figure 3B**, Table). For CD8+ T-cells (**Figures 3C, D**), five of seven subsets analyzed were induced significantly in BCG-RV but not BCG-NRV at prime and six of seven subsets including 3+ effectors sustained after the booster dose (**Figures 3C, D**).

**Figure 4:**
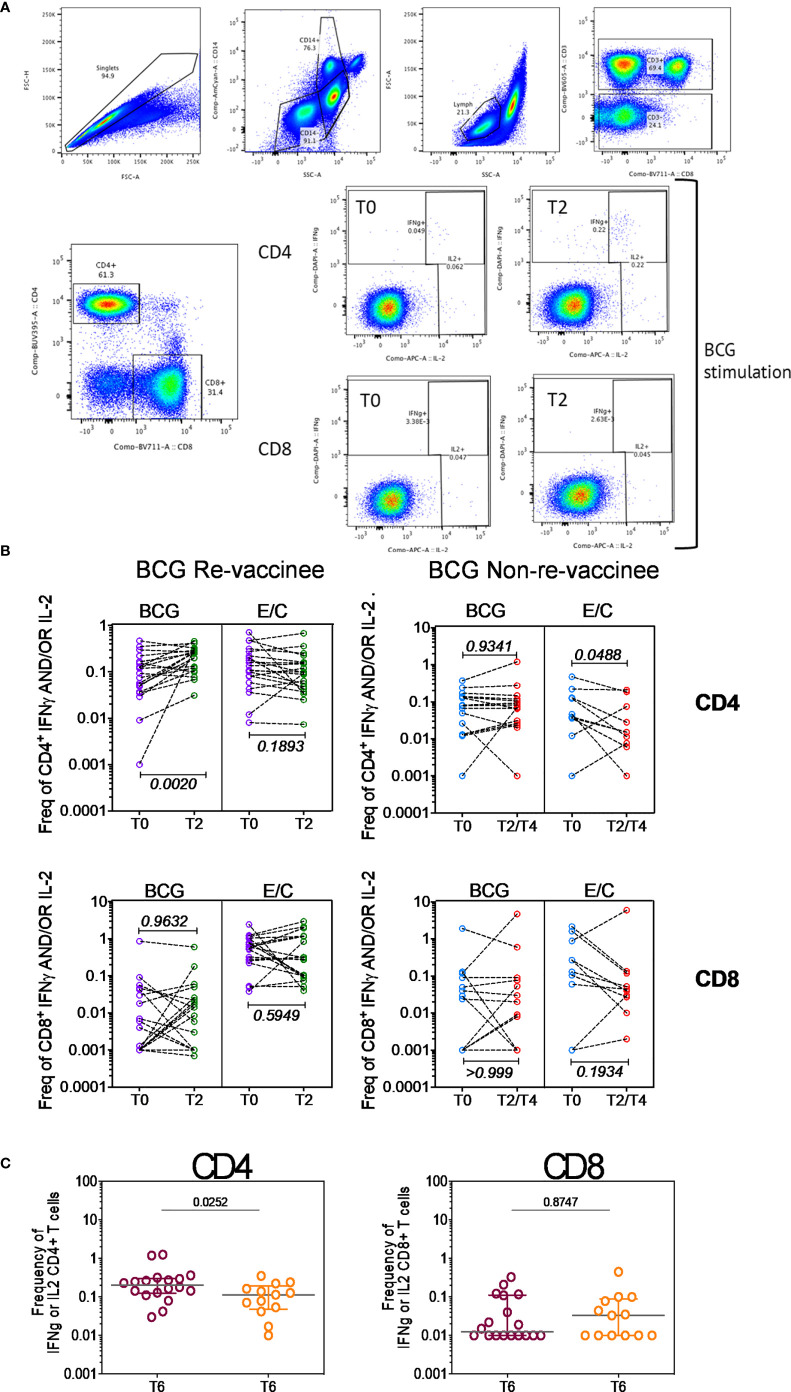
BCG revaccination boosts T cell responses to Mtb antigens at 8-10 weeks post vaccination prior to COVIDSHIELD™ vaccination. **(A)** A representative gating strategy for obtaining CD4+ and CD8+ T cells is shown. Also shown are representative plots for IFN-γ and IL-2 expression post BCG stimulation at T0 and T2 in a BCG re-vaccinee in CD4+ and CD8+ T cells. **(B)** *Mtb*-specific T cell responses after BCG revaccination. Whole blood from 20 BCG-RV and 9 BCG-NRV at baseline (T0) and 10-12 weeks (T2) post-revaccination was stimulated or not with either BCG or ESAT6/CFP10 with for 12 hrs after which samples were subjected to RBC lysis, fixed, frozen and archived. Frozen samples were thawed, washed and stained with a 17-color antibody panel to assess expression of adaptive effectors IFN-γ and IL-2 in the CD4+ and CD8+ T cells. Frequencies of IFN-γ or IL-2 CD4+ and CD8+ T cells after background subtraction were plotted for comparison of responses at T0 and T2. The upper panel shows data for CD4+ T cells (BCG-RV on the left and BCG-NRV on the right) and lower panel shows data for CD8+ T cells (BCG-RV on the left and BCG-NRV on the right). Wilcoxon signed-rank t-test was used for determining statistical significance. **(C)** PBMCs collected from COVISHIELD™ vaccinated BCG-RV (purple circles) and BCG-NRV (orange circles) were stimulated with BCG for 20hr. CD4+ and CD8+ T cells were analyzed for intracellular expression of IFN-γ or IL-2. Grouped scatter plot comparing the frequencies of IFN-γ or IL-2 in CD4+ and CD8+ T cells in samples from COVISHIELD™ vaccinated BCG-RV and BCG-NRV (orange circles) collected at 78-94 weeks post BCG re-vaccination and 20-23 weeks post COVISHIELD™ boost.

Taken together, these data highlight BCG revaccination to significantly enhance the quality of COVISHIELD™ induced T-cell responses, with the most pronounced effect being induction of Spike-specific CD4+ rather than CD8+ T-cell effectors. To understand if this difference is in part explained by inherent differences in the robustness by which BCG revaccination regulates CD4+ versus CD8+ T-cells, we enumerated BCG-specific CD4+ and CD8+ T-cell frequencies following BCG revaccination at 2 time points (**Figure 4A**): first, at T2, where a significant increase in the frequencies of BCG-specific IFN-γ or IL-2 CD4+ but not CD8+ T cells were noted in the BCG-RV but not BCG-NRV group, relative to paired T0 samples (**Figure 4B**). No changes in frequencies of ESAT-6/CFP10 antigen induced CD4 and CD8 T-cells were noted (**Figure 4B**); ESAT-6/CFP-10 being absent from BCG (51), hence serving as a as a negative control. Second, we demonstrate BCG-specific CD4+ T-cell responses to persist until T6 i.e. 78-94 weeks post BCG revaccination (**Figure 4C**), with no change in Mtb-specific CD8 T-cell frequencies between BCG-RV and BCG-NRV, highlighting that BCG revaccination more robustly induced Mtb-specific CD4+ rather than CD8+ T-cell effectors in the timeline studied.

### Unbiased analysis of flow cytometry data extends and confirms a more robust spike-specific memory CD4+ T-cell response in BCG-RV

**Figure 5:**
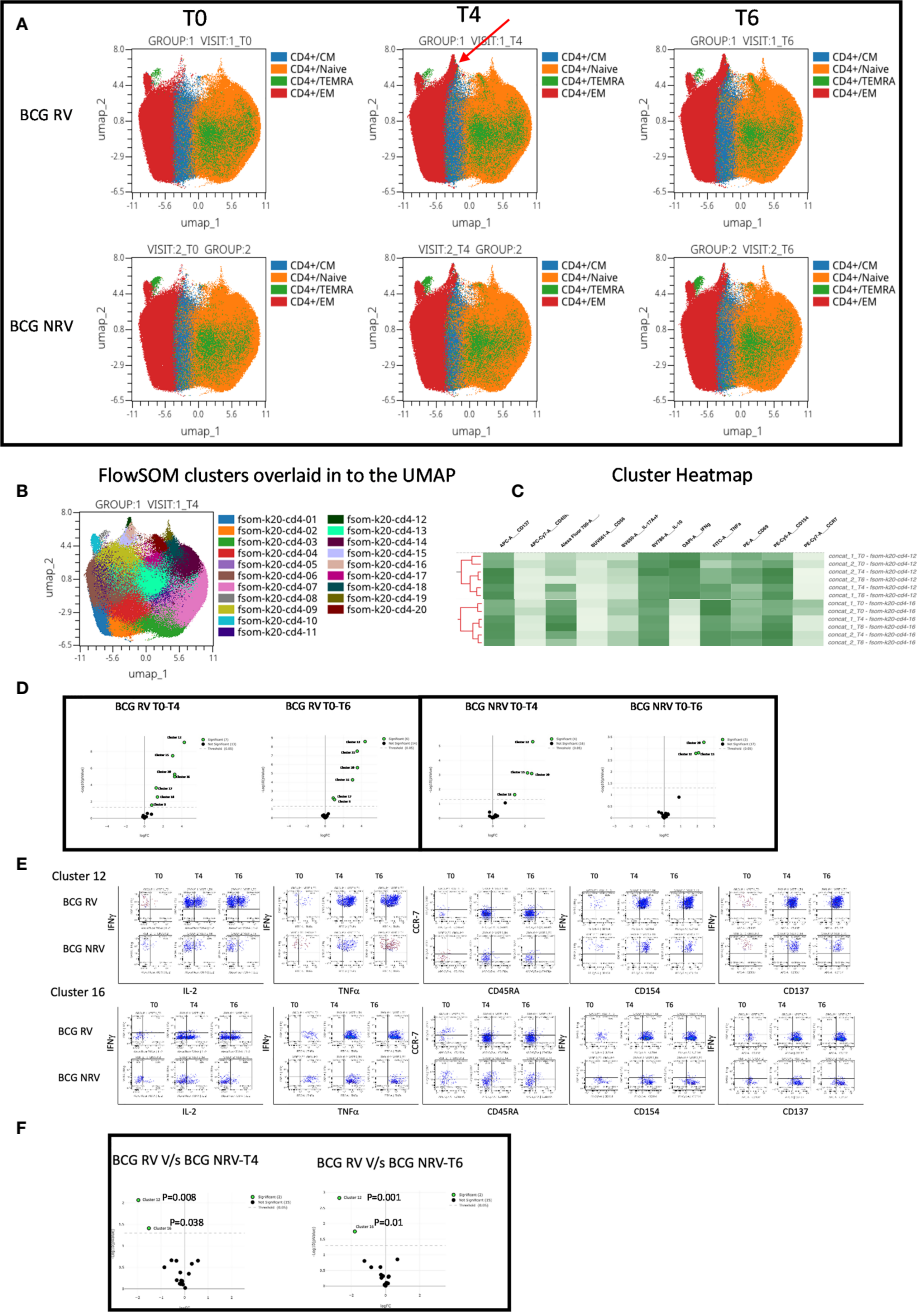
Overview of COVISHILED™ vaccine induced spike specific CD4+ T cell responses in BCG-RV and BCG-NRV subjects measured at baseline (T0), post-prime (T4) and post-boost (T6). **(A)** UMAP of COVISHIELD™ vaccinated BCG-RV and BCG-NRV subjects. **(B)** Unbiased FlowSOM clusters overlaid in the UMAP projection axis. **(C)** Heatmap of cluster 12 and 16 in COVISHIELD™ vaccinated BCG-RV and BCG-NRV subjects. **(D)** Volcano plot of fold change in all FlowSOM clusters in COVISHIELD™ vaccinated BCG-RV and BCG-NRV subjects. **(E)** Bivariate dot plot of Cluster 12 and cluster 16 in COVISHIELD™ vaccinated BCG-RV and BCG-NRV subjects. **(F)** Volcano plot of fold change of all clusters between BCG-RV and BCG-NRV subjects at T4 and T6.

Using OMIQ (http://omiq.ai) we extended and confirmed the above-described manual analysis of flow cytometry data. UMAP analysis of 21 BCG-RV and 13 BCG-NRV longitudinal Spike antigen activated samples highlighted the emergence of a vaccine induced specific CD4 T-cell cluster at T4 (prime) and T6 (boost) time points that was absent at T0 (pre-COVISHIELD™ vaccine) in both BCG-RV and BCG-NRV; identified by an arrow; which were clearly effector memory (EM) T cells (CD45RA and CCR7 negative) (**Figure 5A**). Overlaying UMAP onto FlowSOM identified two clusters (Cluster 10 and Cluster 16 out of a total of 20 clusters, **Figure 5B**) that co-located onto the vaccine-induced cluster identified in **Figure 5A**. Heatmap analysis of these two major clusters across all samples confirmed that the post vaccine and pre-vaccine time points branched distinctly as did samples from BCG-RV and BCG-NRV (**Figure 5C**), with Volcano plots using EdgeR confirming both these clusters to be significantly induced in both BCG-RV and BCG-NRV relative to matched T0 samples (**Figure 5D**); other clusters identified to be significant by EdgeR were deemed minor and not analyzed further. Dot Blot analysis (**Figure 5E**) to deconvolute the cellular subset composition identified Cluster 10 to comprise a dominant IFN-γ expression EM cellular subset that co-expressed IL-2 and TNF-α and AIM markers, CD154 and CD137; Cluster 16 on the other hand comprised an IFN-γ negative EM subset which was predominantly TNF-α positive co-expressing IL-2 and AIM markers. Both clusters 10 and cluster 16 cellular subsets were more abundantly expressed in BCG-RV compared to BCG-NRV at T4 and T6 relative to T0 (**Figure 5E**) with the difference between the two groups confirmed to be significant for both clusters by EdgeR analysis (**Figure 5F**).

**Figure 6:**
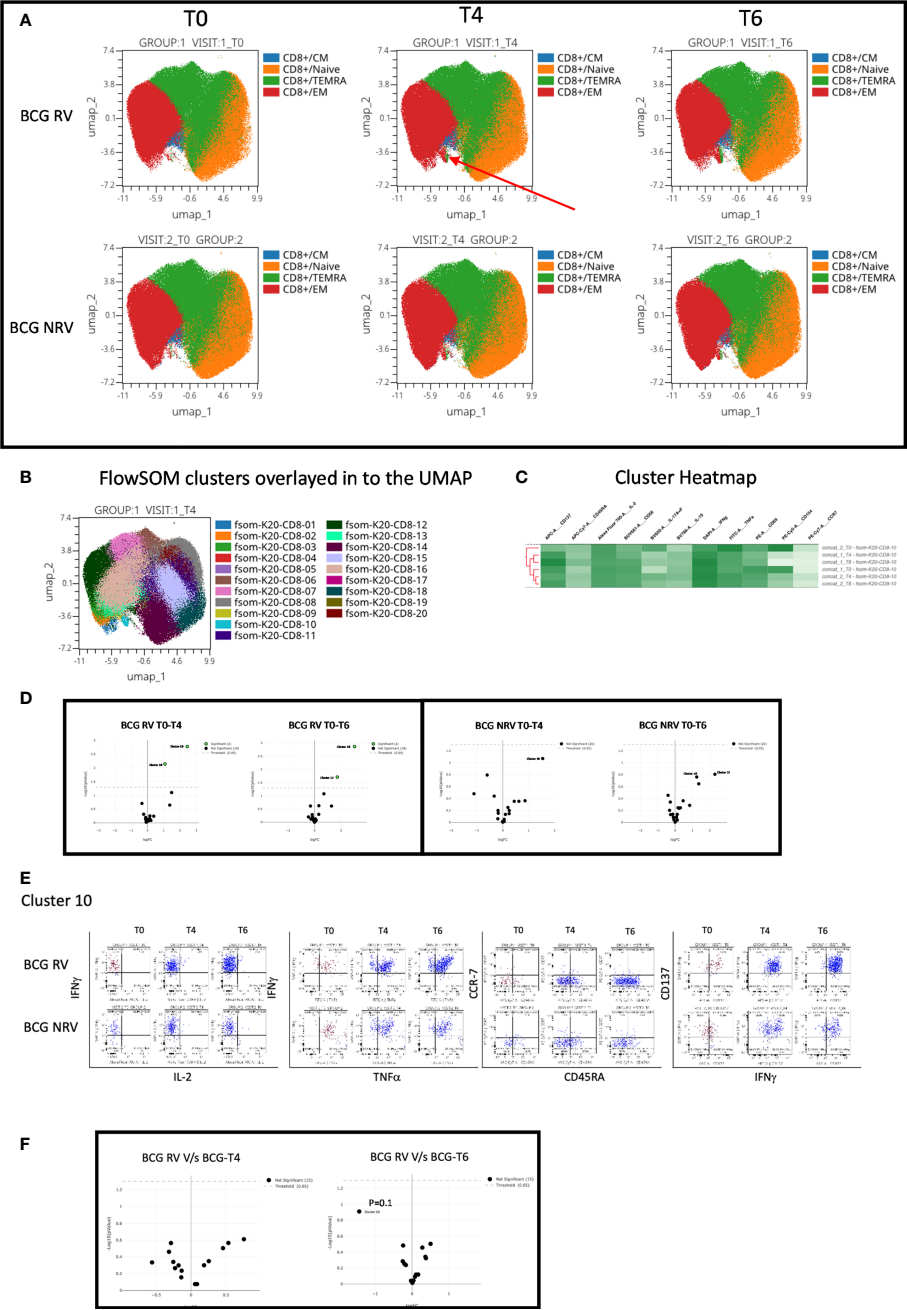
Overview of COVISHILED™ vaccine induced spike specific CD8+ T cell responses in BCG-RV and BCG-NRV subjects measured at baseline (T0), post-prime (T4) and post-boost (T6). **(A)** UMAP of COVISHIELD™ vaccinated BCG-RV and BCG-NRV subjects. **(B)** Unbiased FlowSOM clusters overlaid in the UMAP projection axis. **(C)** Heatmap of cluster 10 in COVISHIELD™ vaccinated BCG-RV and BCG-NRV subjects. **(D)** Volcano plot of fold change in all FlowSOM clusters in COVISHIELD™ vaccinated BCG-RV and BCG-NRV subjects. **(E)** Bivariate dot plot of Cluster 10 in COVISHIELD™ vaccinated BCG-RV and BCG-NRV subjects. **(F)** Volcano plot of fold change of all clusters between BCG-RV and BCG-NRV subjects T4 and T6.

Concomitant analysis of CD8 Spike-specific T-cell responses in **Figure 6**, highlight the following major points: a unique vaccine induced cluster was identified in post-vaccine compared to pre-vaccine samples that comprised a mixture of EM and TEMRA effectors (**Figure 6A**) which colocalized with one major cluster, Cluster 10 (**Figure 6B**); heatmap analysis identified the BCG-RV and BCG-NRV samples to distinctly segregate within this cluster (**Figure 6C**) and for this cluster 10 to be significantly induced in both BCG-RV and BCG-NRV after vaccination (**Figure 6D**). Dot blot analysis identified Cluster 10 to be dominant in IFN-γ and co-expressing TNF-α and CD137 (**Figure 6E**). Though cluster 10 was identified as a significant vaccine -induced CD8 Spike-specific T cell cluster (**Figure 6D**), comparative analysis of BCG-RV and BCG-NRV did not reach statistical significance (**Figure 6F**), confirming that BCG revaccination impacted the quality and quantity of vaccine induced Spike-specific CD4+ T-cell responses more significantly than CD8+ T-cell responses.

### BCG revaccination enhances the breadth of COVISHIELD™ induced immune response

**Figure 7:**
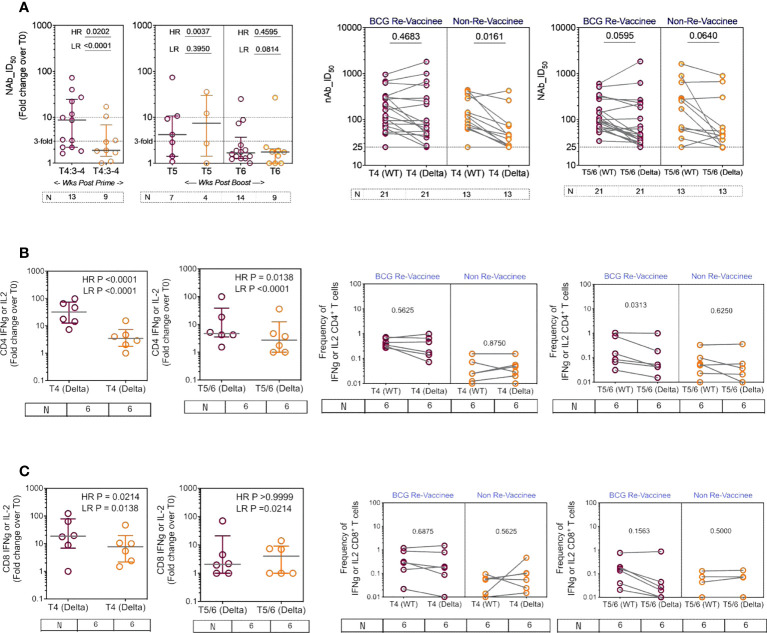
The breadth of the spike response in BCG-RV and BCG-NRV to Wild-type and Delta variant (B1.617.2). Cross-sectional analysis of **(A)** neutralizing antibody (nAb ID_50_) and **(B, C)** CD4+ and CD8+ T cell responses to the Delta variant (B1.617.2) in samples collected from COVISHIELD™ vaccinated BCG-RV (purple circles) versus BCG-NRV (orange circles) at 3-4 weeks post-prime (T4:3-4), 6-7 weeks post boost (T5) and 20-23 weeks post boost (T6). Grouped scatter plots depict the median (horizontal grey line) and interquartile range of fold change in nAb ID_50_ or frequencies of IFN-γ or IL-2 over baseline. Paired line graphs for comparison of **(A)** neutralizing antibody (nAb ID_50_) and **(B, C)** CD4+ and CD8+ T cell responses to the reference wild-type versus Delta variant (B1.617.2) in matched samples collected from COVISHIELD™ vaccinated BCG-RV (purple circles) and BCG-NRV (orange circles) at 2-4 weeks post prime (T4) and 6-7 weeks (T5) or 20-23 weeks (T6) post-boost. PBMCs were stimulated with Spike peptide pool to the delta strain (B1.617.2) and its matched reference WT (0.06 nM) for 20 hr. CD4+ and CD8+ T cells were analyzed for intracellular expression of IFN-γ or IL-2. Statistical significance was determined by Kruskal-Wallis test with Dunn’s correction for line graphs and Wilcoxon matched paired t-test for scatter plots.

We next determined the efficiency with which COVISHIELD™ induced nAb and T-cells specific to spike protein of the Delta variant (B1.617.2). Differences between groups in fold induction of responses over matched baseline values (**Figure 7A**, scatter graphs) were striking: the BCG-RV group had a significantly higher proportion of high and low responders post-prime, but these differences were not sustained with few high responders detected at 20-23 weeks post-booster (**Figure 7A**, scatter graphs). This was also confirmed using a Wilcoxon paired t-test analysis of the nAb response to WT versus Delta strains in each subject (**Figure 7A**, line graphs), which showed that after prime the BCG-RV group had equally efficient nAb to both strains, whereas BCG-NRV had significantly lower nAb to Delta. After the booster, there was a trend for subjects in both groups to have higher nAb to WT, but this difference did not reach significance. These data highlight priming alone induces a more efficient nAb to both WT and Delta in BCG-RV compared to BCG-NRV.

BCG-RV comprised of significantly higher proportion of both CD4 and CD8 high and low responders respectively, highlighting BCG-NRV to be overall weaker T-cell responders to Delta SARS-CoV-2 (**Figures 7B, C**, scatter graphs). Matched analysis of the breadth of CD4+ and CD8+ T-cell responses in a subset of six subjects, to peptides spanning the Delta mutation relative to matched epitopes in the Wuhan strain depict similar T cell responses to both strains in BCG-RV, whilst the magnitude of response was lower in BCG-NRV (**Figures 7B, C**, line graphs). **Figures S6A, B** confirms that Delta-specific nAb and CD4+ or CD8+ T-cell responses correlated significantly. Collectively, above data highlight that BCG-RV mounts more robust spike-specific nAb and CD4+ and CD8+ T-cell responses to the Wuhan and Delta strains compared to BCG-NRV.

### BCG revaccination boosts PAMP-induced effector cytokines in monocytes and PBMCs reflective of enhanced trained immunity

**Figure 8:**
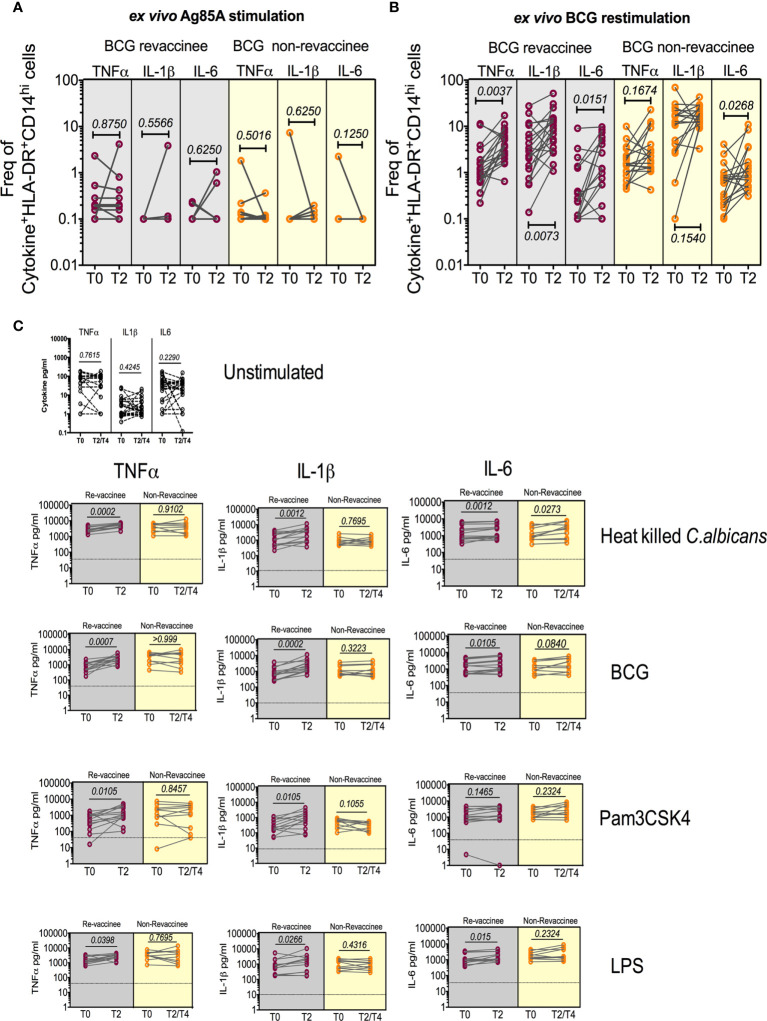
BCG revaccination boosts innate effector responses in HLA-DR+ monocytes and trained immunity effectors to PAMP **stimulation.** Whole blood from 20 BCG-RV and 18 BCG-NRV at baseline (T0) and 10-12 weeks (T2) post-revaccination was or stimulated with either Ag85A **(A)** or BCG **(B)** for 12hr following which samples were frozen. Frozen samples were thawed and stained to assess expression of innate effectors in the monocyte compartment. Frequencies of TNF-α+, IL-1β+ and IL-6+ monocytes after background subtraction were plotted for comparison of responses at T0 and T2. **(C)** PBMC from 13 BCG-RV and 10 BCG-NRV at baseline (T0), 10-12 weeks (T2) or 51-68 weeks (T4) post-re-vaccination were left unstimulated or stimulated with 10^6^ cfu/ml *C. albicans*, 0.2x10^6^ cfu/ml BCG, 1ng/ml LPS and 50μg/ml Pam_3_CSK_4_ for 24hr after which supernatants were harvested for ELISA of TNF-α, IL-1β and IL-6. Absolute concentrations of secreted cytokines were read off a standard curve and plotted after subtraction of background. Cytokines secreted by unstimulated cells (i.e., background) are shown separately at the top of the figure. BCG-RV and BCG-NRV are depicted by grey and yellow shaded areas respectively. Statistical significance was determined by Wilcoxon signed-rank t-test.

BCG reportedly boosts adaptive immune responses to heterologous vaccines by augmenting PAMP-stimulated responses including expression of TNF-α, IL-1β and IL-6, implicated in TI (12). We therefore tested monocyte and PBMC responses to PAMP stimulation in the same subjects probed for COVISHIELD™ induced adaptive responses at baseline and 10-12 weeks post BCG revaccination, a recognized peak time point to measure BCG-induced TI (52), archived prior to the COVID-19 pandemic and prior to COVISHIELD™ vaccination, thus mitigating against SARS-CoV-2 exposure/infection interference. **Figure 8** first shows the frequency of HLA-DR+CD14+ monocyte responses following *in vitro* stimulation with BCG (a recognized PAMP) in a flow cytometry assay (representative staining in **Figure S7**). Parallel cultures with mycobacterial Antigen85A (Ag85A) T-cell peptides served as a negative control (**Figure 8A**). Frequencies of BCG but not Ag85 stimulated HLA-DR+ TNF-α and IL-1β expressing CD14+ cells were significantly higher post BCG revaccination (T2) relative to baseline (T0) in BCG-RV but not BCG-NRVk samples (**Figures 8A, B**), highlighting specificity of BCG induced TNF-α and IL-1β expression. Significant fluctuation of IL-6+ CD14+ monocytes in BCG-NRV samples precluded identification of specific changes in IL-6+. Next, we tested a wider PAMP panel in matched PBMC in a 24hr cytokine secretion assay (**Figure 8C**). The data show TNF-α and IL-1β secretion following stimulation with BCG, *Candida albicans*, Pam_3_CSK_4_ and LPS to be significantly higher at T2 compared to T0 in BCG-RV but not BCG-NRV cultures (**Figure 8C**). Consistent with the flow cytometry data (**Figures 8A, B**), the induction of IL-6 was weaker and fluctuated in BCG-NRV. These data confirm that BCG revaccination can augment innate responses at the monocyte and PBMC level; these early innate changes consistent with enhanced TI in BCG-RV.

### BCG revaccination induces long-term changes in the chromatin accessibility of monocytes

**Figure 9:**
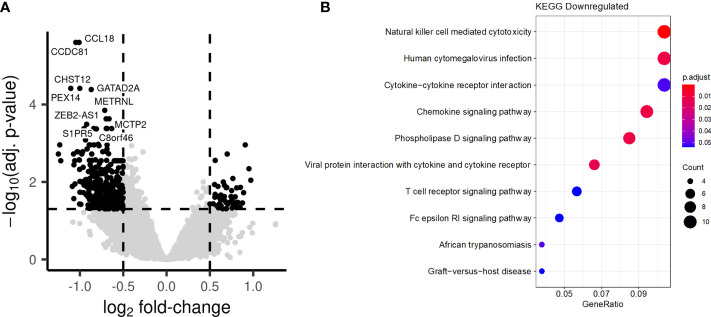
BCG vaccination induces long-term changes in chromatin accessibility. PBMCs were collected from individuals before and after BCG vaccination. **(A)** Comparison of the chromatin accessibility after BCG vaccination to before BCG vaccination. Each point represents a peak. Black points are significantly differentially accessible peaks (FDR<0.05 and absolute log-fold change > 0.5), while grey points do not differ Peaks are labelled by their closest gene by HOMER. **(B)** Barplot showing the most significantly enriched KEGG pathways after BCG. Genes linked to significantly differentially accessible peaks were considered to calculate the enrichment.

Long-term enhancement of the monocyte function by BCG vaccination, which was described as trained immunity, has been reported to be mediated by epigenetic reprogramming of myeloid cells (13). In a final set of experiments, we assessed the chromatin accessibility of PBMCs isolated before (T0) or after BCG vaccination at T2 by ATAC-seq (Assay for Transposase-Accessible Chromatin using sequencing). A large number of loci displayed significant differences in accessibility of chromatin after BCG vaccination, with both higher and lower accessibility being observed (**Figure 9A**). Interestingly, while pathway analysis did not identify biological processes in which genes had a significant increase in chromatin accessibility, we observed an enrichment in several pathways among the genes with decreased accessibility, linked to chemokine signaling (**Figure 9B**).

## Discussion

This study provides the first in-depth analysis of immune responses to COVISHIELD™, the most widely distributed COVID19 vaccine in India, combined with novel insights into the beneficial adjuvant effects of prior BCG vaccination on subsequent COVISHIELD™ induced immune responses. Our study is distinct from ongoing clinical trials testing impact of BCG vaccination on SARS-CoV-2 infection outcome. Three important and unique strengths support our conclusions: (i) the inclusion of a control group: we were able to compare the COVISHIELD™ induced Ab and T cell responses in both BCG-RV and BCG-NRV similar for age, BCG vaccination at birth and time post COVISHIELD™ vaccination (ii) the ability to probe COVISHIELD™ induced responses without interference of infection: all subjects included were seronegative at baseline with no history of SARS-CoV-2 infection; further, sampling after COVISHIELD™ prime was before the widespread second COVID-19 wave in India, also reflected in COVISHIELD™ prime immune response kinetics being similar to a primary immune response in seronegative populations (53–57). (iii) Importantly, we could integrate data from multiple adaptive and innate immune assays on the same set of longitudinal samples compared to pre-pandemic, pre-BCG and pre-COVISHIELD™ vaccine baseline samples, thereby establishing unambiguous immune assay cut-off values.

BCG did not alter the kinetics of the COVISHIELD™ Ab or T cell response, but significantly impacted its quality in three major ways. First, Ab and T cell responses were significantly higher in BCG-RV, including a greater proportion of high responders. Some BCG-RV individuals had exceptionally high T cell responses (>10-fold change) that persisted for 20-23 weeks post-boost; such high responses were not detected or declined sharply in BCG-NRV. This heterogeneity may be intrinsic to the COVISHIELD™ vaccine (58) and potentially amplified by BCG. Our data highlights BCG revaccination to synergize with COVISHIELD™ to amplify vaccine-specific Ab and T-cell responses as well as enhance the durability of the induced immune response. Second, BCG promoted the induction of polyfunctional T-cell responses, a characteristic that ascertains vaccine efficacy against chronic viral infections (59), including SARS-CoV-2 where vaccine-induced multifunctional T cells correlate with enhanced protection from emerging variants (60). Interestingly, one study showed polyfunctional T-cells to be enhanced following the ChAdOx1 nCoV-19 vaccine booster indicating the booster may serve to enhance the quality and not just magnitude of a vaccine-induced response (60). We contend that significant induction of polyfunctional spike-specific T-cells after prime and their persistence after the booster can potentially contribute to the heterologous benefit of BCG. Third, BCG-RV produced a more robust response to the Delta mutant of SARS-CoV-2 highlighting greater breadth of immune responses, a function that was globally, including India, associated with milder disease in ChAdOx1 nCoV-19 vaccinees (61–63). With a strong correlation noted between nAb and T-cells specific for both the Wuhan and Delta strains, we contend that BCG vaccination has the potential to expand the breadth of the Ab and T cell response against SARS-CoV-2 variants of concern.

The double-blind, randomized placebo-controlled trial in Malawi showed no benefit of BCG revaccination on all-cause mortality (64). However, the immediate or short-term beneficial effects of BCG may have been masked primarily by deaths due to non-infectious causes and the long gap between deaths and BCG vaccination during follow-up. Notably, the choice of genetically different BCG strains has been linked to the highly variable efficacy against tuberculosis and all-cause mortality in clinical trials and may also impact the effectiveness of BCG against COVID-19 (16, 65). Hence, off-target effects observed upon vaccination with BCG-Russia (TUBERVAC™) strain used in this study can vary with that of BCG-Glaxo strain used in Malawi (64, 65). Our data is consistent with previous work highlighting the benefit of prior or synchronous BCG vaccination in boosting heterologous vaccine responses (35), and consistent with the results of the Mexican study that demonstrated prior BCG vaccination to enhance the Pfizer/Biotech induced nAb response, 4 weeks after BCG vaccination (37). In the context of COVID-19, BCG may not be unique and is consistent with emerging acceptance of the benefits of heterologous vaccination strategies. It’s been noted that immunization with existing vaccines such as the Influenza, OPV, MMR, Varicella Zoster in the recent past (≤ 5 years) can confer protection against SARS-CoV-2 by reducing infection rates, improving clinical outcomes and/or boosting nAbs induced during infection (66). Indeed, heterologous prime-boost immunization regimens per se maybe more beneficial, i.e., ChAdOx1 nCoV-19 and mRNA-1273 or ChAdOx1 nCoV-19 and BioNTech have been shown to augment COVID-19 vaccine efficacy by enhancing spike-specific IgG, nAbs as well as CD4+ and CD8+ T-cells including robust recognition of variants of concern above levels induced by homologous vaccination (67–71).

One important mechanism by which BCG vaccination can boost heterologous vaccine responses is its intrinsic PAMP characteristics and ability to regulate innate immunity. Individuals in our study who showed boosted adaptive responses to COVISHIELD™ also exhibited evidence of trained immunity 8-12 weeks post BCG revaccination (**Figure 8**) in terms of enhanced TNF-α and IL-1β secretion upon *in vitro* PAMP re-stimulation. These immunological effects were accompanied by long-term changes in chromatin accessibility as assessed by ATAC-seq, with multiple loci displaying either increased or decreased accessibility (**Figure 9**). These findings support the concept that induction of trained immunity is accompanied by epigenetic changes in innate immune cells (72). In addition, previously published studies in animals and humans have shown that enhanced adaptive responses often follow the induction of trained immunity by BCG, suggesting that TI can indeed impact the adaptive arm of the immune system (8, 32, 73). The relevance of these findings has been earlier demonstrated by studies showing that vaccination with BCG increases the resistance of experimental animals to subsequent vaccinia virus infection and this was mediated *via* the CD4+ T cell response (73). Similarly higher Th1 and Th17 cytokine levels in addition to innate responses were observed to *in vitro* stimulation with *Staphylococcus aureus* and *Candida albicans* in PBMC from individuals vaccinated with BCG (8). Also, volunteers who received BCG prior to influenza vaccination had signatures of trained immunity as well as augmented anti-H1N1 humoral responses (32).

Our observation that the heterologous benefit of BCG was more evident on COVISHIELD™ induced spike-specific CD4+ rather than CD8+ T cells is consistent with BCG as a recognized inducer of Th1 CD4+ T cell effectors through three potential mechanisms: firstly, trained monocytes have higher expression of MHC-II and costimulatory molecules CD80 and CD86 making them better antigen presenting cells (74, 75); secondly, trained monocytes have a higher expression of PRRs like CD14, TLR4 and mannose receptor and produce more pro-inflammatory cytokines such as TNF-α and IL-1β which can enhance T cell responses (8) and thirdly, cytokines secreted by trained monocytes e.g., IL-1β and IL-6 are key drivers of Th differentiation to Th1, Th17 or ex-Th17- subsets (76–78). Apart from these suggested mechanisms, it has been speculated that BCG vaccination might lower thresholds for T cell activation on account of the cytokine milieu that exists due to primed/trained monocytes (79). It should be noted that boosted T cell responses to COVISHIELD™ in individuals vaccinated with BCG might also be due to presence of cross-reactive epitopes in BCG and SARS-CoV-2 vaccines (80). Importantly, SARS-CoV-2 specific CD4+ T cell responses too have been demonstrated to be more robust compared to the CD8+ T cell response in the context of vaccination as well as infection (81, 82).

Our data has important healthcare implications despite a small sample size imposed by COVID-19 lockdown restrictions. Moreover, since this is an observational study the control group was not administered with the placebo. Our study was designed to test whether immune responses induced by highly efficacious COVISHIELD™, can be further boosted in a SARS-CoV-2 infection and vaccine naïve population. The fact that BCG does have this potential in a young healthy population calls for further analysis on timing/dose/nature of prior BCG vaccination on heterologous vaccine responses in the elderly and the immunocompromised versus testing if pre-existing COVID-19 vaccine or SARS-CoV-2 infection induced responses can be enhanced by subsequent BCG vaccination. We highlight the potential of using a cheap and globally available vaccine as an adjuvant for novel and emerging vaccines, an area of significant scientific interest (83, 84), with the added advantage that the timeline over which BCG adjuvant effects have been noted span several years (32, 34). We postulate this to be linked to BCG leaving an imprint on innate cells/responses combined with its ability to induce long lasting mycobacterial antigen specific CD4+ memory T-cells which can provide T-T and T-B cell benefit, a concept highlighted by 1980’s work showing Ab responses induced by a foreign antigen coupled to tuberculin being significantly higher in BCG pre-sensitized animals (85). We call for further studies to understand heterologous benefits of BCG and the associated impact of tuberculosis prevalence on COVID-19 vaccine immunity.

## Data availability statement

The ATAC Seq data presented in the study are deposited in the EGA repository, accession number EGAS00001006417.

## Ethics statement

The studies involving human participants were reviewed and approved by institutional ethics review committee of St. John Medical College Hospital, Bangalore, IEC Ref no: (IEC/1/896/2018). The patients/participants provided their written informed consent to participate in this study.

## Author contributions

MM and AV conceived the BCG revaccination project. AV conceived the aims of this study. SRak, AA, VA, and AV designed the experiments. SRak, VA, and AA performed the immunology experiments and analyzed the data. VA helped with flow cytometer instrument set up, acquisition, and data analysis. CP performed in-house antibody binding assay and analyzed the data. TM and KD performed neutralization assay. SB performed LIAISON SARS-CoV-2 TrimericS IgG assay. VA and NC helped with processing of blood from clinical cohorts. GD’S, MD, and PDw oversaw clinical recruitment. SS and SRao collected clinical samples, provided the patient details, and wrote the clinical methodology for the manuscript. VA, KM, and SJ analyzed the flowcytometry data using OMIQ. BG, MN, MZ, and YL were involved in the epigenetic assessments. SRak, AA, VA, and AV wrote the manuscript. SD reviewed ICS assay data. SD, MM, SB, KD, GD’S, MD, PDas, TM, SJ, KM, MN, and AV edited the manuscript. All authors contributed to the article and approved the submitted version.

## Funding

This work was principally funded by DBT Grant Reference No.BT/PR30219/MED/15/189/2018 as part of a joint DBT-NIH award to AV, MM, KDS and SD. We acknowledge additional funding from DBT-BIRAC (BT/COVID0073/02/20) to AV. This work was also supported by the National Institute of Allergy and Infectious Diseases of the US National Institutes of Health (UM1 AI 068618).

## Acknowledgments

The authors thank the volunteers for participating in this study and acknowledge the contributions of clinical research workers at St John’s Research Institute. We acknowledge Professor Vijaya Satchidanandam, Indian Institute of Science for support. We acknowledge the Malcolm Coptcoat Trust for its small consumable grant. We thank the IISc Central FACS facility for help with flow cytometry. We thank Dr David Moyes, King’s College London for the kind gift of heat-inactivated *Candida albicans*.

## Conflict of interest

The authors declare that the research was conducted in the absence of any commercial or financial relationships that could be construed as a potential conflict of interest.

## Publisher’s note

All claims expressed in this article are solely those of the authors and do not necessarily represent those of their affiliated organizations, or those of the publisher, the editors and the reviewers. Any product that may be evaluated in this article, or claim that may be made by its manufacturer, is not guaranteed or endorsed by the publisher.

## Supplementary material

Supplementary Figure 1 In-House Ab binding assays correlation with nAb. **(A)** Plasma SARS-CoV-2 anti-Spike, RBD and Nucleocapsid (N) protein IgG titres measured by an in-house ELISA in COVISHIELD™ vaccinated subjects measured overtime. IgG titres at baseline (T0), 2-4 weeks post-prime (T4), 6-7 weeks (T5) and 20-23 weeks (T6) post-boost. **(B)** Correlations between SARS-CoV-2 anti-Spike, RBD and Nucleocapsid protein IgG titres and corresponding neutralizing antibody responses (nAb ID50) in COVISHIELD™ vaccinated subjects measured overtime. **(C)** Correlations between SARS-CoV-2 IgG titres measured by LIAISON^®^ SARS-CoV-2 TrimericS IgG assay and corresponding neutralizing antibody responses (nAb ID50) in COVISHIELD™ vaccinated subjects measured overtime.

Supplementary Figure 2 Representative FACS Gating Strategy. Schematic representation showing sequential gating strategy for analysis of CD4+ and CD8+ T-cells in PBMC. **(A)** Representative flow cytometry plots of spike-specific CD4+ and CD8+ T cells after 20hr stimulation with spike peptide pool compared to negative control (Unstimulated) at T0 (baseline), T4 (post-prime) and T5/6 (post-boost). **(B)** Representative examples from two COVISHIELD™ vaccinees.

Supplementary Figure 3 PBMCs were stimulated with Spike peptide pool (0.06 nM) for 20 hr. CD4+ and CD8+ T cells were analyzed for intracellular expression of IFN-γ, IL-2 and TNF-α. Grouped scatter plot with median (horizontal grey line) and interquartile range comparing the frequencies of IFN-γ, IL-2 and TNF-α in **(A)** CD4+ and **(B)** CD8+ T cells over baseline or at 2-4 weeks post-prime (T4), 6-7 or 20-23 weeks post boost (T5/T6). Statistical significance was determined by Kruskal-Wallis test with Dunn’s correction for line graphs.

Supplementary Figure 4 COVID vaccination does not boost responses to SARS-CoV-2 M, N and CEFT. CD4+ and CD8+ T cell responses to SARS-CoV-2 M, N and CEFT in COVISHIELD™ vaccinated subjects measured overtime. PBMCs from individuals collected from COVISHIELD™ vaccinated BCG-RV (purple circles) and BCG-NRV (orange circles) at baseline (T0), 2-4 weeks post-prime (T4), 6-7 weeks (T5) and 20-23 weeks (T6) post-boost were stimulated with peptide pools specific to SARS-CoV-2 membrane, nucleocapsid proteins (0.06nM) and CEFT (1ug/ml) for 20hr. CD4+ and CD8+ T cells were analyzed for intracellular expression of IFN-γ or IL-2. Line graphs comparing the frequencies of IFN-γ or IL-2 in CD4+ and CD8+ T cells in samples are shown. Kruskal-Wallis test with Dunn’s correction was used for determining statistical significance between longitudinal timepoints.

Supplementary Figure 5 The vaccine induced nAb and T cell responses correlate in BCG-RV and BCG-NRV. Correlations between IFN-γ and/or IL-2 expression in **(A)** CD4+ and **(B)** CD8+ T cells and corresponding neutralizing antibody responses (nAb ID50) in COVISHIELD™ vaccinated BCG-RV (purple circles) and BCG-NRV (orange circles) measured overtime.

Supplementary Figure 6 Correlations between neutralizing antibody and T cell responses to the SARS-CoV-2 delta strain in BCG-RV and BCG-NRV. IFN-γ or IL-2 expression in **(A)** CD4+ (left panel) and **(B)** CD8+ (right panel) T cells and corresponding nAb ID_50_ to the delta variant in COVISHIELD™ vaccinated BCG-RV (purple circles) and BCG-NRV (orange circles) measured overtime.

Supplementary Figure 7 Representative gating strategies. A representative gating strategy for obtaining HLA-DR+CD14hiCD16- monocytes are shown. Also shown are representative plots for TNF-α, IL-1β and IL-6 expression in the HLA-DR+CD14hiCD16- population in one *ex vivo* unstimulated and BCG stimulated sample from a BCG-RV at T0 and T2.
