# Supplementary material for: Evidence for the heterologous benefits of prior BCG vaccination on COVISHIELD™ vaccine-induced immune responses in SARS-CoV-2 seronegative young Indian adults
Source: Front Immunol. 2022 Oct 4;13:985938. doi: 10.3389/fimmu.2022.985938 (PMC9577398; doi:10.3389/fimmu.2022.985938)
Supplement: Supplementary Figure 1 — In-House Ab binding assays correlation with nAb. (A) Plasma SARS-CoV-2 anti-Spike, RBD and Nucleocapsid (N) protein IgG titres measured by an in-house ELISA in COVISHIELD™ vaccinated subjects measured overtime. IgG titres at baseline (T0), 2-4 weeks post-prime (T4), 6-7 weeks (T5) and 20-23 weeks (T6) post-boost. (B) Correlations between SARS-CoV-2 anti-Spike, RBD and Nucleocapsid protein IgG titres and corresponding neutralizing antibody responses (nAb ID50) in COVISHIELD™ vaccinated subjects measured overtime. (C) Correlations between SARS-CoV-2 IgG titres measured by LIAISON® SARS-CoV-2 TrimericS IgG assay and corresponding neutralizing antibody responses (nAb ID50) in COVISHIELD™ vaccinated subjects measured overtime. [file Presentation_1.pptx]

## Slide 1
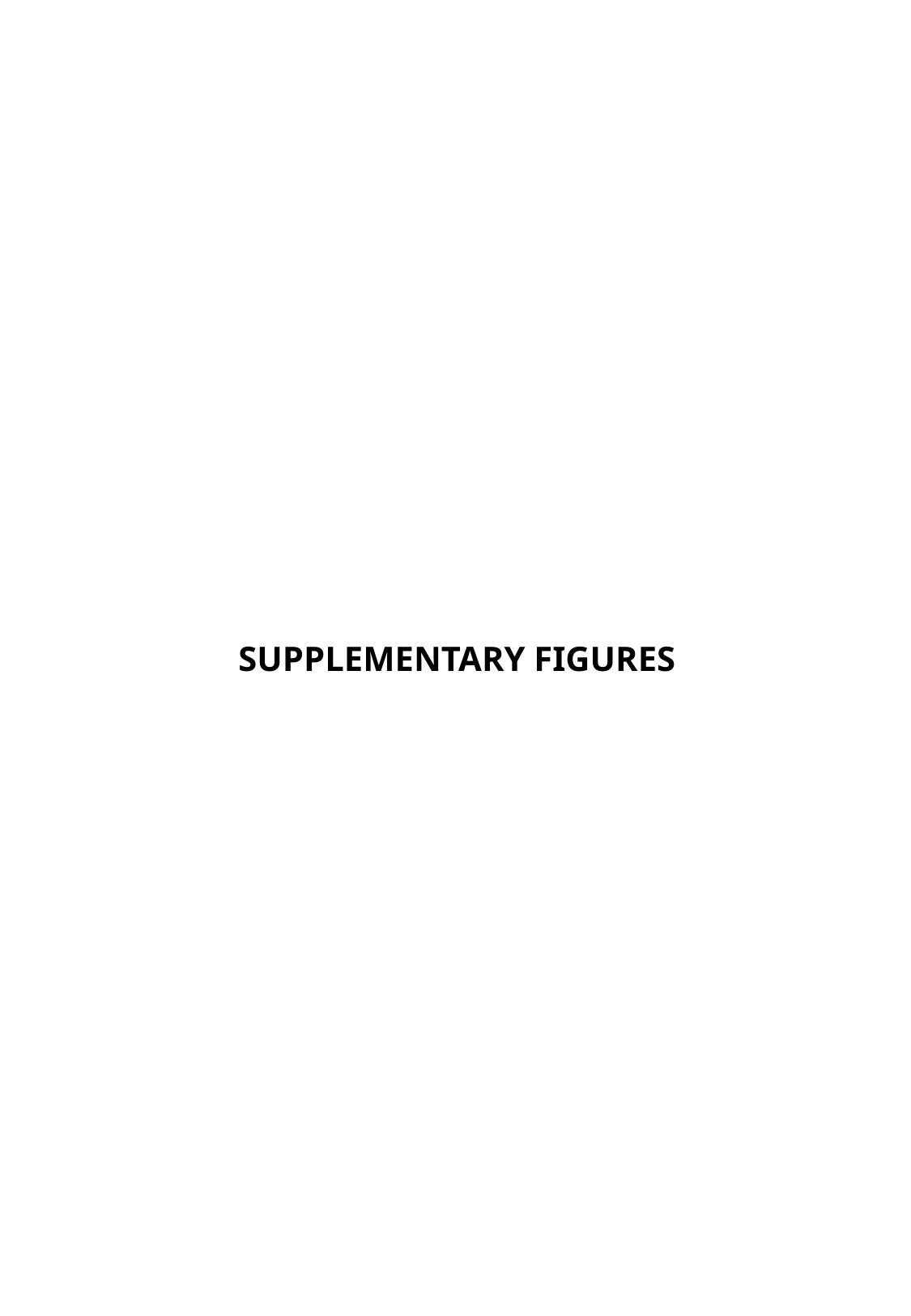

SUPPLEMENTARY FIGURES

## Slide 2
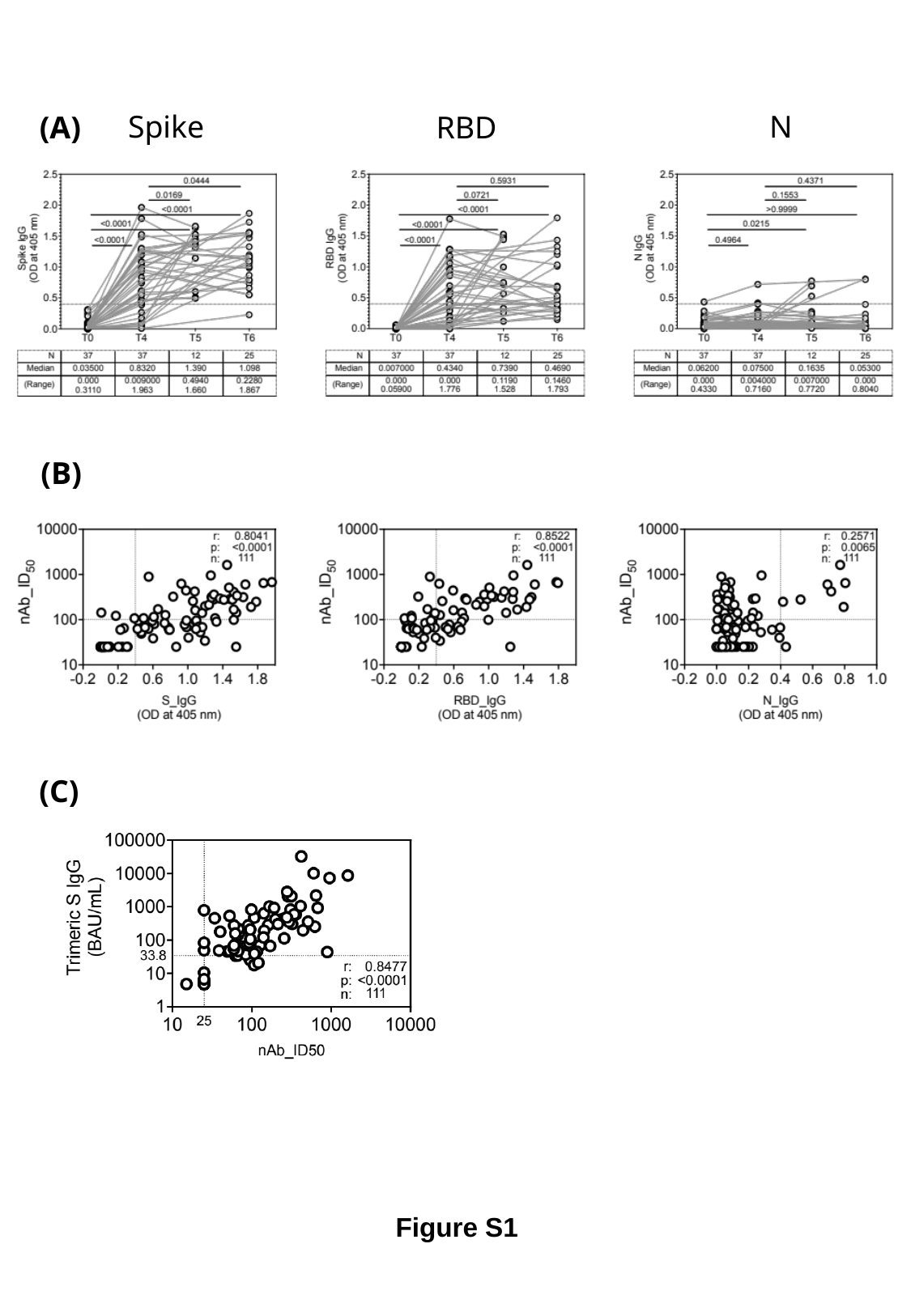

Spike
N
(A)
RBD
(B)
(C)
Figure S1

## Slide 3
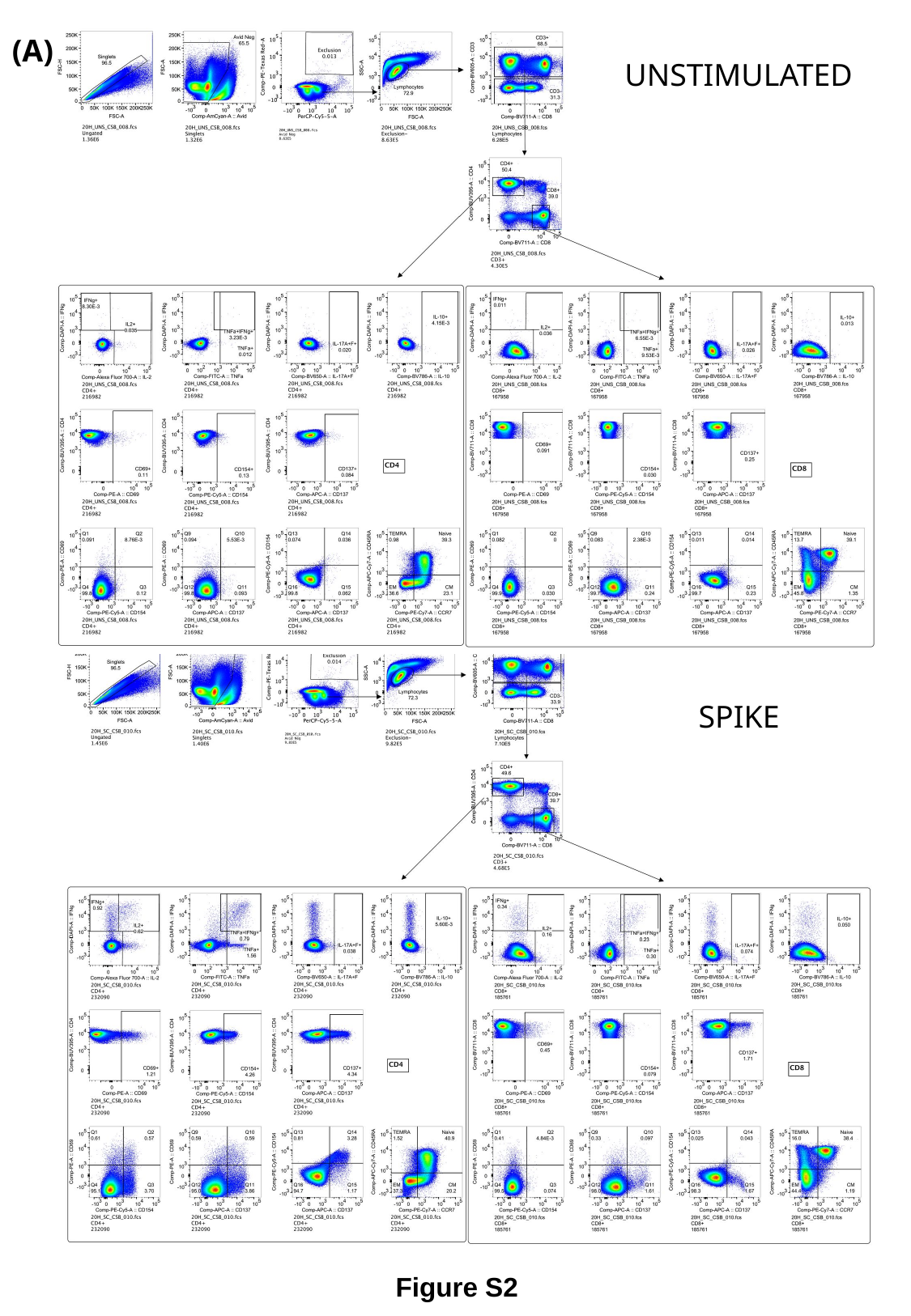

(A)
UNSTIMULATED
SPIKE
Figure S2

## Slide 4
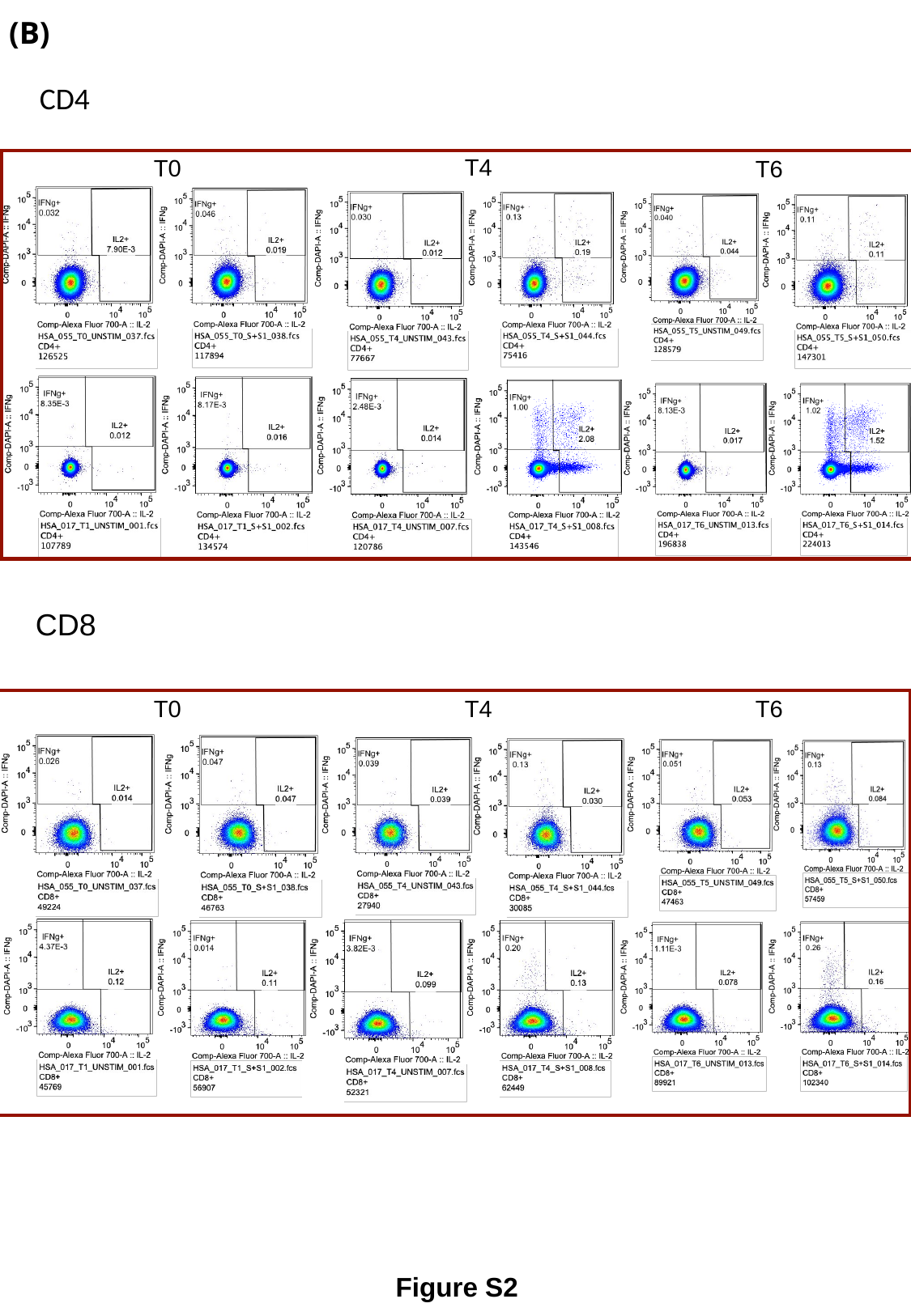

(B)
CD4
T4
T0
T6
CD8
T0
T4
T6
Figure S2

## Slide 5
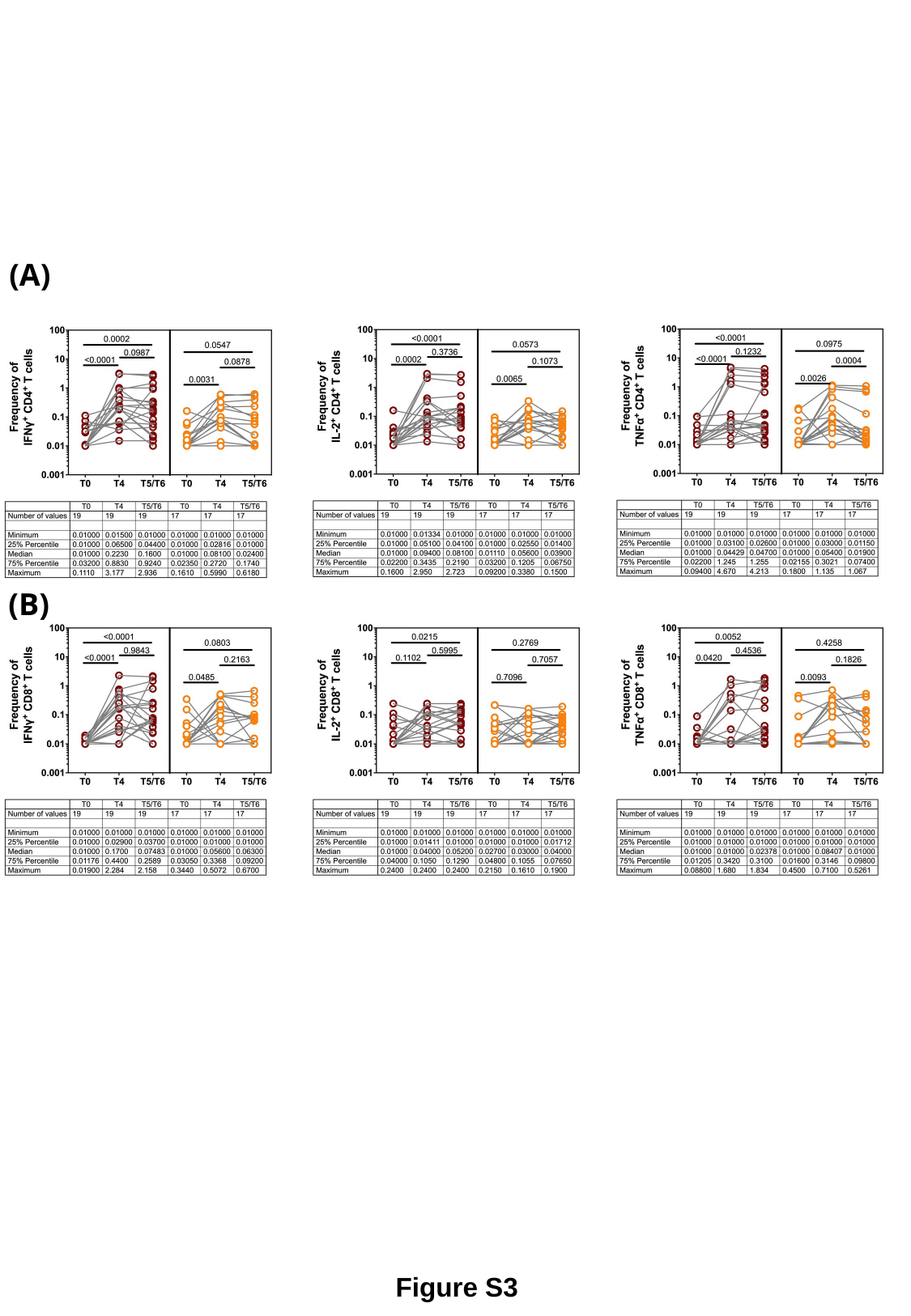

(A)
(B)
Figure S3

## Slide 6
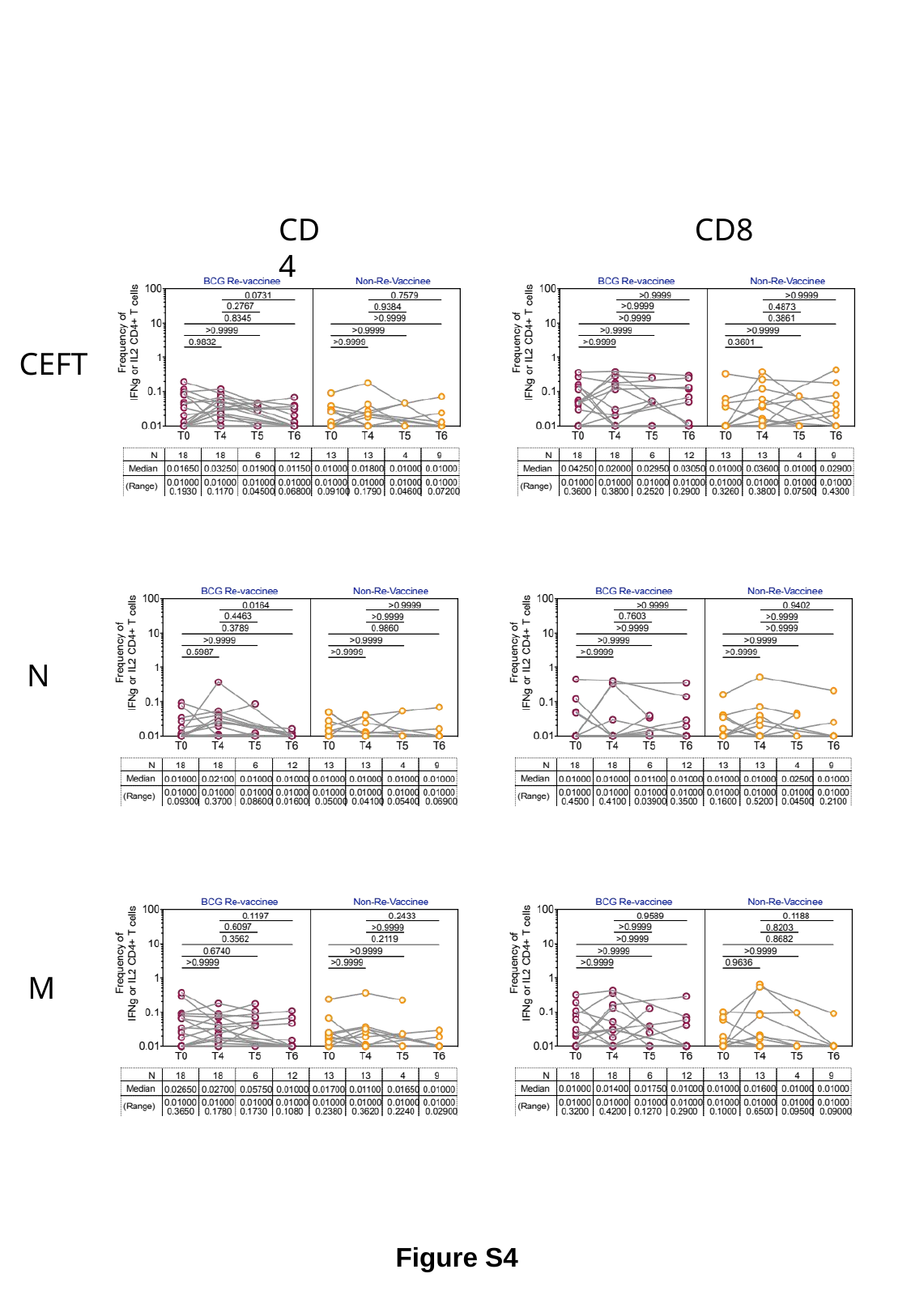

CD4
CD8
CEFT
N
M
Figure S4

## Slide 7
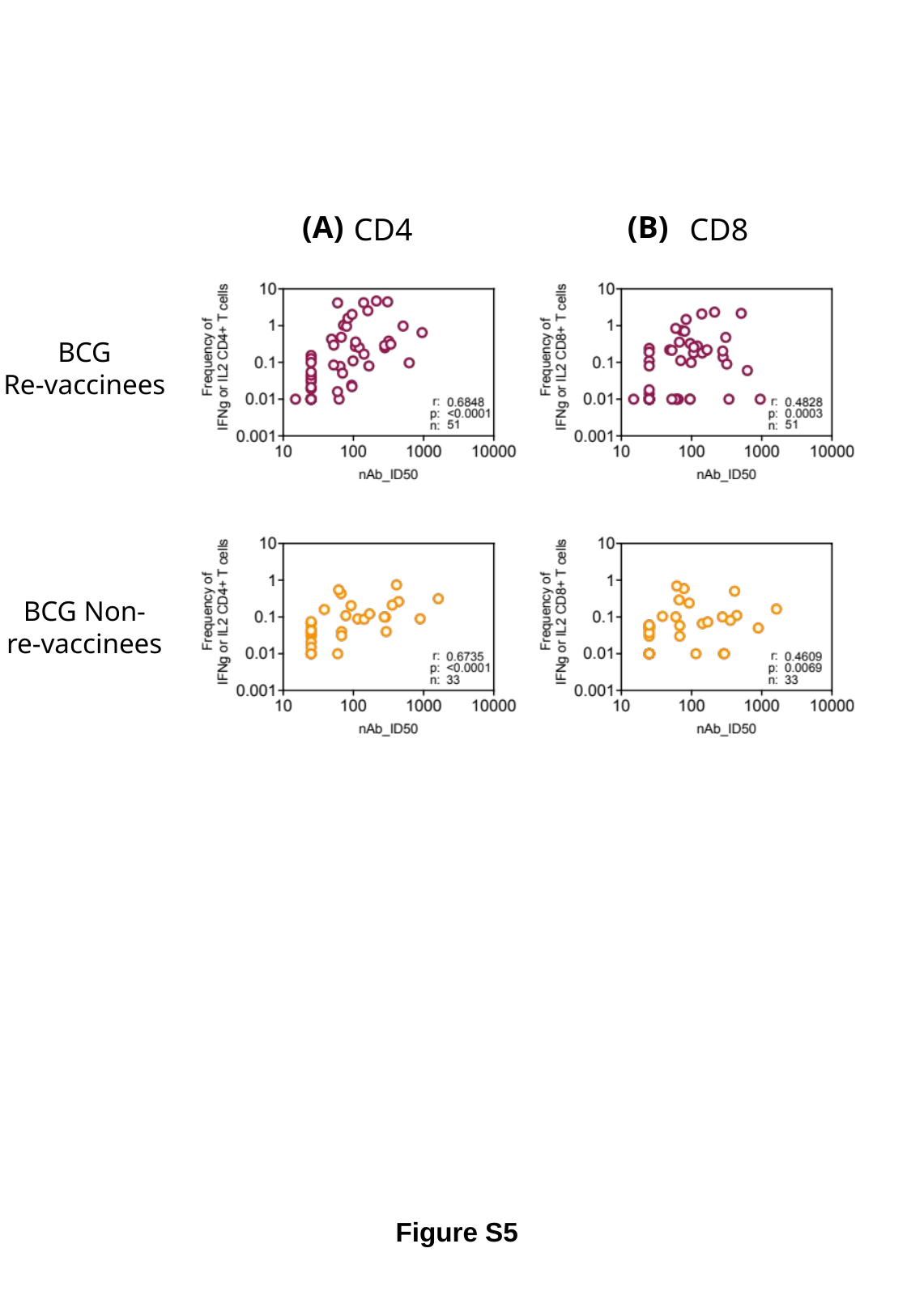

(A)
(B)
CD4
CD8
BCG
Re-vaccinees
BCG Non-
re-vaccinees
Figure S5

## Slide 8
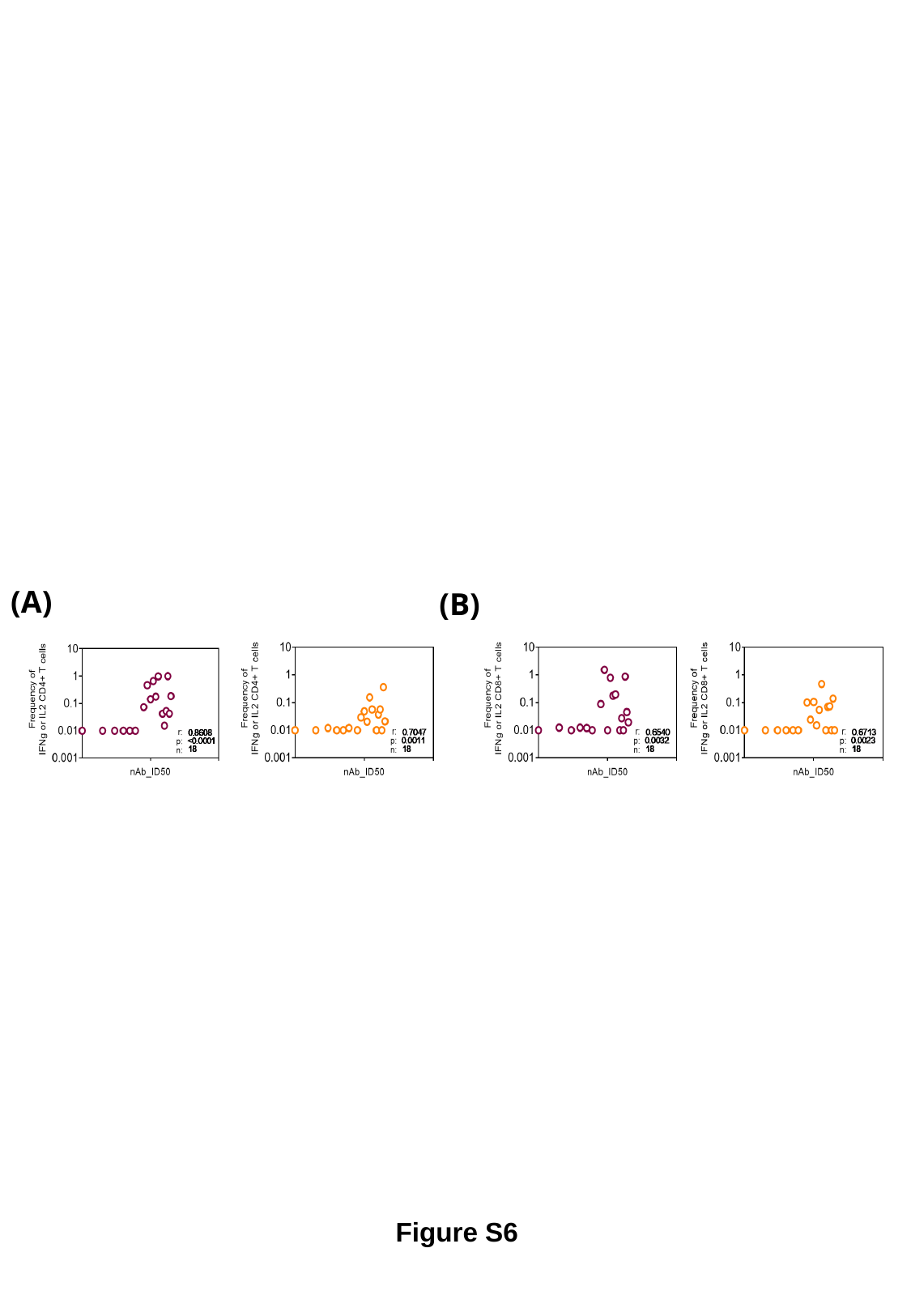

(A)
(B)
Figure S6

## Slide 9
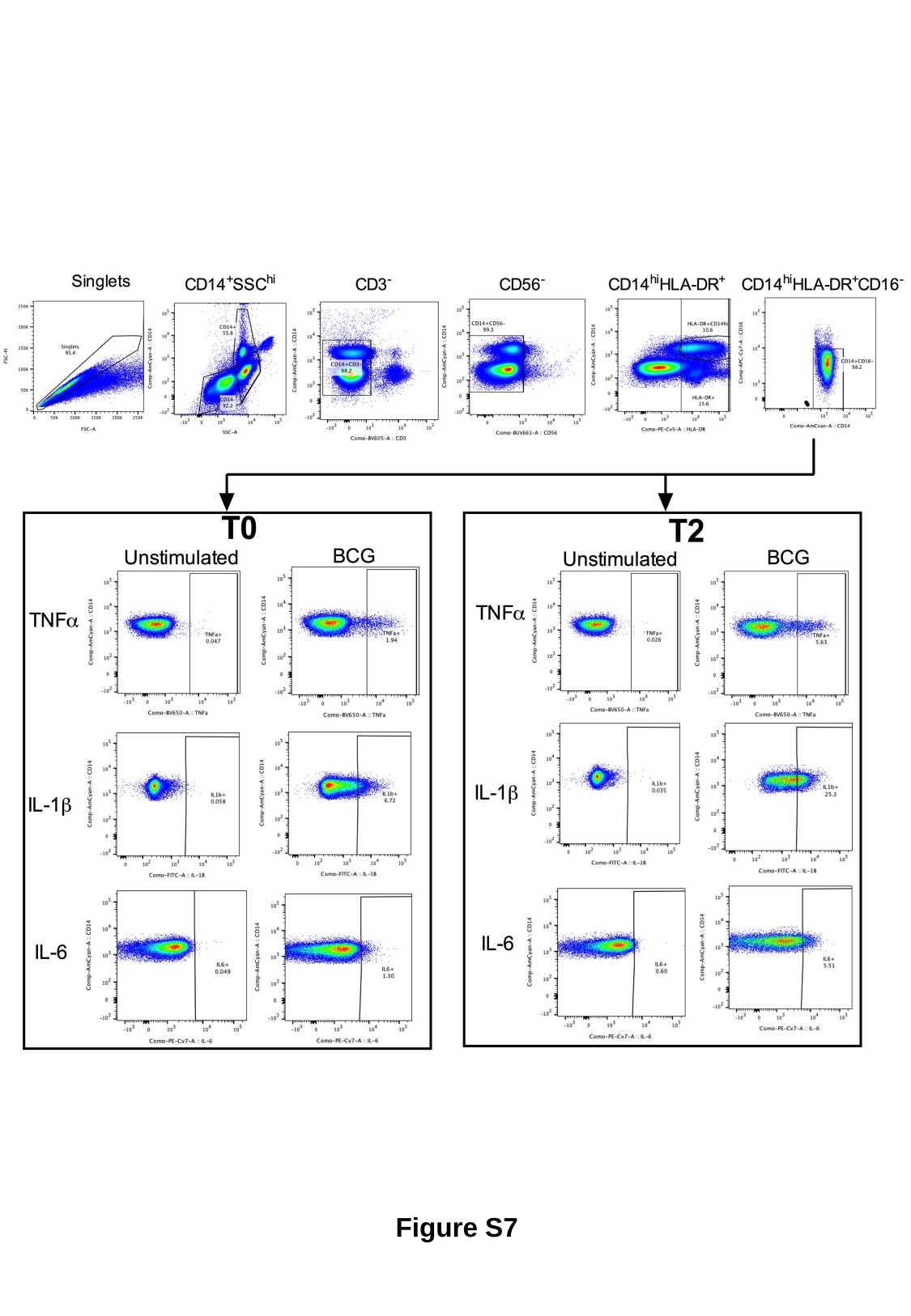

Figure S7
